# Nitrogen oxides concentration and emission change detection during COVID-19 restrictions in North India

**DOI:** 10.1038/s41598-021-87673-2

**Published:** 2021-05-07

**Authors:** Prakhar Misra, Masayuki Takigawa, Pradeep Khatri, Surendra K. Dhaka, A. P. Dimri, Kazuyo Yamaji, Mizuo Kajino, Wataru Takeuchi, Ryoichi Imasu, Kaho Nitta, Prabir K. Patra, Sachiko Hayashida

**Affiliations:** 1 Research Institute for Humanity and Nature, Kyoto, Japan; 2 Japan Agency for Marine-Earth Science and Technology, Yokohama, Japan; 3 Graduate School of Science, Tohoku University, Sendai, Japan; 4 Radio and Atmospheric Physics Lab, Rajdhani College, University of Delhi, New Delhi, India; 5 School of Environmental Sciences, Jawaharlal Nehru University, New Delhi, India; 6 Kobe University, Kobe, Japan; 7 Meteorological Research Institute, Japan Meteorological Agency, Tsukuba, Japan; 8 Institute of Industrial Science, The University of Tokyo, Tokyo, Japan; 9 Atmosphere and Ocean Research Institute, The University of Tokyo, Chiba, Japan; 10 Faculty of Science, Nara Women’s University, Nara, Japan

**Keywords:** Environmental chemistry, Environmental impact, Environmental impact, Atmospheric chemistry

## Abstract

COVID-19 related restrictions lowered particulate matter and trace gas concentrations across cities around the world, providing a natural opportunity to study effects of anthropogenic activities on emissions of air pollutants. In this paper, the impact of sudden suspension of human activities on air pollution was analyzed by studying the change in satellite retrieved NO_2_ concentrations and top-down NOx emission over the urban and rural areas around Delhi. NO_2_ was chosen for being the most indicative of emission intensity due to its short lifetime of the order of a few hours in the planetary boundary layer. We present a robust temporal comparison of Ozone Monitoring Instrument (OMI) retrieved NO_2_ column density during the lockdown with the counterfactual baseline concentrations, extrapolated from the long-term trend and seasonal cycle components of NO_2_ using observations during 2015 to 2019. NO_2_ concentration in the urban area of Delhi experienced an anomalous relative change ranging from 60.0% decline during the Phase 1 of lockdown (March 25–April 13, 2020) to 3.4% during the post-lockdown Phase 5. In contrast, we find no substantial reduction in NO_2_ concentrations over the rural areas. To segregate the impact of the lockdown from the meteorology, weekly top-down NOx emissions were estimated from high-resolution TROPOspheric Monitoring Instrument (TROPOMI) retrieved NO_2_ by accounting for horizontal advection derived from the steady state continuity equation. NOx emissions from urban Delhi and power plants exhibited a mean decline of 72.2% and 53.4% respectively in Phase 1 compared to the pre-lockdown business-as-usual phase. Emission estimates over urban areas and power-plants showed a good correlation with activity reports, suggesting the applicability of this approach for studying emission changes. A higher anomaly in emission estimates suggests that comparison of only concentration change, without accounting for the dynamical and photochemical conditions, may mislead evaluation of lockdown impact. Our results shall also have a broader impact for optimizing bottom-up emission inventories.

## Introduction

Movement restrictions or ‘lockdowns’ emerged worldwide as the most popular government regulation in 2020 to control COVID-19 transmission. It induced a natural experiment on the global scale with spillover social^1^, economic^1,2^ and environmental impacts^3–5^. Early lockdown in January and February in China resulted in decreased economic activities, revealing a decline in regional pollutants. Nitrogen oxides (NOx ≡ NO + NO_2_), notorious for their direct and indirect health impacts^6^, gained additional importance because of the natural experiment. As NO_2_ is less prone to long-range transport owing to its short life-time (2–7 h), its concentration changes during the COVID-19 lockdowns are being probed to obtain a clear estimation of the regional impact of local policy actions. For example, NO_2_ concentrations in Chinese cities decreased by up to 50% to 60% as measured by ground monitors^7,8^, and Ozone Monitoring Instrument (OMI) and TROPOspheric Monitoring Instrument (TROPOMI) satellite retrieved columns^4,9^. Strong declines in NO_2_ were also observed in Europe, South Korea, and the United States^5,10^. Indian cities, which went under varying intensities of ‘lockdown’ severity since March 25, 2020 too reported large reductions in NOx concentrations^11,12^. About 60% to 90% decline in surface NOx concentrations was observed in Delhi during the first week of lockdown compared to the pre-lockdown week^13,14^. A review of change in pollutant concentration across Indian cities as measured by ground monitors is summarized elsewhere^11,15^.

One question of interest is the reduction in NOx concentrations which is attributable to the COVID-19 lockdown restrictions^4,10,11,16^. This is challenging because the short-term NO_2_ concentrations are regulated not only through the anthropogenic emissions but also non-linearly through meteorology, atmospheric chemistry and soil NOx emission. On a long-term time-scale (order of years), trends in NO_2_ concentrations are associated with gradual changes in anthropogenic emissions (from industries and vehicles) due to the technological upgradation, economic growth and emission regulations^17^. However in a very short term (order of few days to weeks), NO_2_ concentrations have an inverse linear relationship with boundary layer height and wind speed within the boundary layer due to upliftment of air mass^18^. At seasonal scale, changes in concentration are exhibited primarily due to the seasonal variation of NO_2_ lifetime. The lifetime is governed by the chemistry of hydroxyl radical (OH) and photolysis under changing solar zenith angle, and the meteorological processes like dispersal, wet and dry deposition.

Yet, the most common method to evaluate the impact of lockdown on NO_2_ has been to take a statistical analysis comparing the air quality during and before the lockdown or with the same periods in previous years^14,16^. Such evaluations failed to consider the effect of some or all of the above confounders on the concentrations. Only a handful of studies have systematically disentangled the impact of lockdown on the NOx emissions from the concentrations by considering the effect of wind fields on pollutant transport, for example by simulating wind trajectories^19–21^, employing machine learning-based deweathering technique^22^, applying chemical transport model (CTM) to determine seasonal and meteorological effects^23^.

A major challenge to enable evaluation of the impacts of short-term interventions is the estimation of emission changes^22^. To that end, recent remote sensing based top-down NOx emission estimation approaches are promising^24–26^. For instance, Miyazaki et al.^20^ assimilated data from multiple satellites and in-situ observations into a CTM to evaluate COVID-19 related emission changes in China.

**Figure 1:**
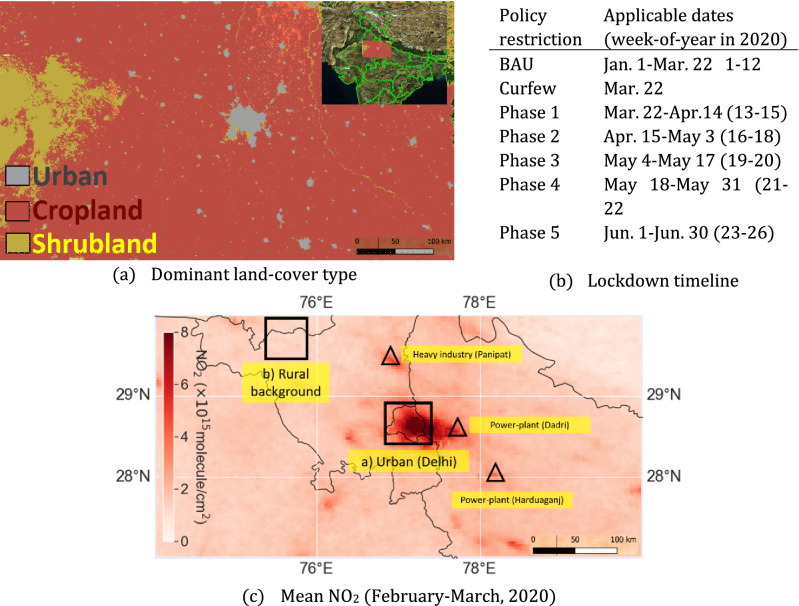
(**a**) Dominant land cover types in the study domain (74°E, 27°N—80°E, 30°N) are urban areas, croplands and desert shrublands. (**b**) Timeline of lockdown policies, where BAU refers to business-as-usual conditions (for details see Table S1). (**c**) Mean NO_2_ column density (TROPOMI) during February 1 to March 20, 2020. Representative location of an urban area (Delhi) and rural background (Fatehabad) is shown in black box, in addition to other prominent emission sources (power-plant at Dadri and Harduaganj and an industrial cluster at Panipat) marked in a triangle. Figures generated using ‘Cartopy’ version 0.16 and ‘Rasterio’ version 1.2 modules of Python 3.6 (https://www.python.org/downloads/release/python-360/).

In light of the above-mentioned concerns in assessing the impact of lockdown on NOx, the present work has two-fold objectives: (a) estimate concentration changes robustly, and (b) estimate top-down emission changes. We focused on a polluted region in northern India (shown in Fig. 1), that underwent one of the largest nation-wide continuous lockdowns (spanning 74 days from March 25, 2020 to June 7, 2020). The advantage of our evaluation of COVID-19 impact on NOx is that while the concentration change estimates takes in account the effect of seasonality and long-term changes in NO_2_ concentration, the emission change estimates disentangle the effect of wind field meteorology using ‘steady-state continuity equation based model’^26^. We assessed the weekly trends in concentration in the context of 2015–2020 satellite records as well as the top-down NOx emissions for 2019 and 2020.

## Methodology

### Study location and policy restrictions

The study domain is defined over a region (74°E, 27°N–80°E, 30°N) in the Indo-Gangetic Plains (IGP) as shown in Fig. 1. The domain is notable for its poor air quality, dense settlements and polluting industries. It includes diverse point- and area-based emission sources such as coal-based power-plants at Dadri (location: 77.61°E, 28.60°N; capacity: 2600 MW), Harduaganj (location: 78.13°E, 28.01°N; capacity 610 MW) and Jhajjar (location: 76.35°E, 28.49°N; capacity: 1320 MW), densely populated urban Delhi (urban center: 77.21°E, 28.60°N), smaller cities such as Meerut (urban center: 77.70°E, 28.98°N), Mathura (urban center: 77.67°E, 27.49°N), Aligarh (urban center: 78.07°E, 27.87°N), highway network radiating outward from the center, brick-kilns^27^ and rural regions not lying downwind of emission sources (sample region bounded by 75.38°E, 29.38°N–75.13°E, 29.90°N). In the domain, climatic conditions vary from a mean low temperature of 3 °C during winter (December to February) to a high of 45 °C during the pre-monsoon summer (March—June). The region has also seen long-term trends and seasonality in NOx concentrations^28,29^.

Prior to March 22, 2020 business-as-usual (BAU) conditions prevailed as there was no movement restriction pertaining to COVID-19. The lockdown was implemented over India in several phases starting from March 25 (week 13) and lasting till May 31. Phase 1 of the lockdown consisted of the strictest restrictions followed by relaxation of restriction intensities in subsequent phases. From June 1, 2020 the lockdown was gradually rolled back (Phase 5). The timeline of the lockdown-phases with regards to restrictions on the common emission sources is shown in Table S1.

### Datasets

#### *OMI and TROPOMI retrieved tropospheric NO*_*2*_* column*

Tropospheric NO_2_ column retrievals were available from the OMI on-board Aura satellite^30^ and the TROPOMI on-board Copernicus Sentinel-5 Precursor satellite^31^. NO_2_ from OMI (henceforth referred to as OMI NO_2_) is available since 2004 at a pixel size of approximately 13 × 24 km^2^. While offline and near-real time NO_2_ from TROPOMI (henceforth referred to as TROPOMI NO_2_) is available at a higher spatial resolution of 6 × 7km^2^, its data record only goes back until recently (since mid-2018). Both OMI NO_2_ and TROPOMI NO_2_ follow a similar tropospheric NO_2_ column retrieval algorithm. First the slant columnar densities (SCD) are retrieved by differential optical absorption spectroscopy (DOAS) fitting of the backscattered radiance to lab-measured absorption spectra of atmospheric gases such as NO_2_, ozone, water vapor and others. From the SCD, tropospheric vertical column densities (VCD) are isolated by removing the stratospheric contribution and applying air mass fractions^32^. Typically, OMI and TROPOMI retrieve NO_2_ at approximately 13:30 (local time). Over India, the difference in tropospheric NO_2_ from OMI and TROPOMI is generally random. In comparison with OMI (‘OMNO2′ level 2 version 3.1 product), the bias of TROPOMI (offline level-2 product) is $- { }0.2 \pm 0.8 \times 10^{15}$(1σ) ${\text{molec}}/{\text{cm}}^{2}$, which is − 6% ± 21%(1σ) in relative value^33^.

OMI NO_2_′s level-3 daily gridded product (‘OMNO2d’ version 3) with a cloud fraction less than 30% and filtered for ‘row anomaly’ (see Section S2), was downloaded from NASA’s Giovanni portal^34^. From the Google Earth Engine cloud-platform^35^, TROPOMI’s tropospheric NO_2_ (‘tropospheric_NO2_column_number_density’ band of ‘S5P_OFFL_L2_NO2′ product) was cloud screened (by discarding pixels with effective cloud fraction greater than 20%) and downloaded. The daily concentration (from Monday to Sunday) datasets were aggregated to weekly mean concentration to overcome the periodic weekly cycle of increase (on weekdays) and decrease (on weekends) in NO_2_ concentrations^36^. For the study domain, the relative difference of NO_2_ concentrations between 2020 and 2019 from the OMI and the TROPOMI was similar and within 5% of each other (see Section S3).

#### Gridded meteorological data

Meteorological fields from National Center for Environmental Prediction (NCEP) FNL (Final) Operational Global Analysis and Forecast gridded datasets^37^ are available every 6 h at a 0.25° × 0.25° spatial resolution. Horizontal wind field vectors ($w$), namely zonal (*u*) and meridional (*v*) components, at 80-m altitude were downloaded for 2019 and 2020. *w* was used for estimating NO_2_ flux. To ensure the temporal compatibility of the wind vectors with the satellite datasets for analysis, the meteorological datasets were interpolated to 13:30 local time. Wind vectors at a shallow elevation were considered to represent the advection of emission plumes close to the surface, such as vehicles and factories. The time series of meteorological parameters over Delhi (77°E, 28.5°N) is shown in Fig. S2.

#### Activity data

Detailed emission activity data forms critical component of emission inventories. As updated or real-time estimates of emission activity are seldom available, indicators of emission causing activities, such as electric power production reports from thermal power plants or community mobility behavior can serve as reliable proxies for trends in emission activities^38^. Pearson correlation between the time-series trends of such proxy indicators with the satellite retrieved NO_2_ concentration and the top-down NOx emission were estimated to assess the representativeness of ground activity by the concentrations and emissions.

Daily total electricity production reports for each power-plant across India are available from the Power System Operation Corporation Limited^39^. These reports were used as a proxy for daily emissions from the major coal-based thermal power plants situated in the study domain: Dadri and Harduaganj (location shown in Fig. 1). Community mobility behavior in the form of the percentage reduction in daily mobility patterns from the baseline levels are available from Google’s ‘Community Mobility Reports’^40^ since February 2020, aggregated for administrative states. The defined baseline period represents median value of mobility from the 5‑week period January 3 to February 6, 2020 across different categories of places. Its trend was used. Out of the available patterns in Community Mobility Reports, ‘workplace related travel’ behavior reduction in Delhi was used as a proxy for vehicular activity and effectively the aggregate vehicular emission, based on the assumption that it represents the most common vehicle usage in Delhi.

### Concentration change estimation

The analysis flowchart of the study is summarized in Fig. S1. To robustly evaluate the NO_2_ concentration change during the lockdown over Indian cities in Indo Gangetic Plain (IGP), we need to establish the expected concentration, $C_{B}$, that would have existed had the trends typical of previous years prevailed in 2020. Consideration of such trends is necessary to establish $C_{B}$ due to the seasonality and long-term trends (on the order of years) in NOx concentrations in the domain^28,29^. $C_{B}$ was calculated by obtaining the annual seasonality and long-term trend^28^ of concentration (from 2015 to 2019) over the study location and then extrapolating the trend to 2020.

There are several methods to decompose a time-series into its seasonal and long-term components such as ‘seasonal and trend decomposition’ (STL)^41^, fast Fourier transformation, regression fitting, and others. Due to the relative ease with which the model parameters can be specified and interpreted, STL was used here for the component decomposition. Our choice of dataset to perform STL was guided by the availability of OMI longer albeit spatially coarser temporal record of NO_2_ concentration $C_{o}$. The low frequency annual trends and the high frequency seasonal cycles were decomposed from the weekly mean NO_2_ concentration $C_{o}$ retrieved from 2015 to 2019. To ensure that STL decomposition was not affected by outlying pollution episodes, concentrations higher than 99.5 percentiles were discarded. Box-cox transformation was performed on the concentration prior to STL decomposition to ensure they are normally distributed. Pollution episodes that are non-periodic or random interventions could not be decomposed statistically and were discarded as residues.

Under the assumption that the business-as-usual conditions typical of 2015 to 2019 had prevailed, the seasonal and annual components were extrapolated to another year (to 2020) to obtain the expected concentration $C_{B} \left( t \right)$ for each week. As in long-term, NO_2_ concentrations are proportional to emissions, extrapolating the long-term concentration trend implicitly considers the emission trends. The extrapolation was performed using the ‘exponential trend smoothing’ (ETS) function available in R language. ETS extrapolation was repeated using bootstrap sampling with 1000 draws to get the confidence interval of the predictions. Due to the heavy computation required for each STL decomposition and ETS extrapolation, $C_{B} \left( t \right)$ were calculated only over urban Delhi and a background rural region (location bounds are specified in the previous Section). Concentration change over these two locations was evaluated by comparing $C_{B} \left( t \right)$ with $C_{o} \left( t \right)$ of 2020.

As TROPOMI has a limited temporal record, its NO_2_ concentration could not be used to assess concentration change through the robust approach described above. It was used for analyzing the spatial gradients of differences between weekly mean TROPOMI NO_2_ concentrations in 2019 and 2020.

### Top-down emission change retrieval

NOx is a key tropospheric urban air pollutant that is produced mainly from fossil fuel combustion. Its primary emission mostly takes place in the form of NO which is then oxidized through chemical and photolytic pathways to NO_2_, collectively referred to as NOx. Several techniques exist for deriving NOx emissions using satellite retrieved columnar concentrations, such as (a) optimizing the available emission inventories with satellite derived top-down constraints on NOx emissions^24^, (b) inversely fitting a dispersion model on the concentrations over the affected region to derive emissions from the source region^25,42^ or (c) applying mass-conservation based steady-state continuity equation model^26^. (a) presents a compromise between the high spatial resolution of TROPOMI and the coarser model simulation, especially close to strong emission sources^42^. (b) requires a careful definition of the region affected by plume advection, by choosing the product of wind-speed and the NOx lifetime^42^. Emissions thus calculated can be overestimated should the NOx be influenced by other strong sources outside or inside the region^42^. In practice such conditions are sometimes hard to meet over linear or area-based emission sources, for example in Fig. 1, advected plumes can be seen overlapping with downwind emission sources.

With such challenges, (c) was adopted to estimate emissions from multiple point-sources. As (c) primarily depends on the adequacy of the wind vectors, it does not require defining a region around each emission plume (unlike dispersion models) and is useful for estimating emissions at each pixel. Our approach is inspired by the implementation of the continuity equation by Beirle et al.^26^, to relate the divergence ($D$) of NOx emission flux with the sink ($S$) and emission ($E$) as $D = E - S$. The total emission flux along the meridional and zonal wind field, $w,$ is given as $LCw$, where $C$ is the TROPOMI NO_2_ concentration , and $L$ is the ratio of NOx and NO_2_ concentration. The daytime NOx sink,$S$, is constituted by the reaction of NO_2_ with OH to form HNO_3_^43^, Assuming a first order reaction of NO_2_ with the time constant $\tau$ (the average lifetime of NOx in the boundary layer), $S$ is given as $LC/\tau$. Thus $E$ can be calculated as Eq. (1).

1 $$E = \frac{LC}{\tau } + \nabla \left( {LCw} \right)$$

$\tau$ depends on factors like the photolysis rate, length of night, OH concentrations and meteorological conditions such as wind, relative humidity and temperature^36^. It is of the order of one day but varies by about one order of magnitude with time and place. In tropical regions $\tau$ varies from 7 h in January to about 4 h in July^24^. Since the domain lies in a subtropical and semi-arid region, $\tau$ was regressed linearly between 7 to 4 h depending on the day of the year. $L$ represents the partitioning of NOx into NO and NO_2_ in the polluted layer by serving as a surrogate for NO to NO_2_ chemistry^44^. It depends on ozone concentration, NO_2_ photolysis rate and temperature. Close to the surface, NOx is present as NO_2_ except when close to strongly emitting sources where conversion of NO to NO_2_ is limited by ozone concentration. A typical value of $L$ as 1.32 was assumed for the urban noon-time pollution conditions^26,43,45^.

Theoretically the divergence term, $D\left( { = \nabla \left( {LCw} \right)} \right)$ should be positive over emission sources and negative elsewhere due to the chemical sink. Following Eq. 3, if $S\left( { = LC/\tau } \right)$ is adequately specified, then it numerically compensates the negative $D$ resulting in a net zero emission rate $E$ over non-emission source field. However, if $S$ is underestimated such as by assuming $\tau$ larger than actual lifetime, we could incorrectly obtain negative $E$. As the $\tau$ of 4 h was derived for pollution on top of the tropospheric background concentrations^46^, the sink term $S$ would be biased high in absence of the background correction. A background correction was applied for the NO_2_ concentration by removing the 5th percentile of all concentration in the domain^26^. Daily meridional and zonal fluxes were stacked and mean averaged for each week after discarding the pixels flagged with clouds masks. The sum of divergence of weekly mean flux and the sink of weekly mean NO_2_ concentration was used to estimate weekly emission rate over each TROPOMI pixel.

Emission rates over urban Delhi and power-plants at Dadri and Harduaganj were estimated and compared with activity reports. A region spanning 50 × 50 km^2^ (16 × 16 TROPOMI pixels) around urban Delhi, and 20 × 20km^2^ (6 × 6 TROPOMI pixels) regions around Dadri and Harduaganj power-plants were selected. The region for calculating emission rate was chosen to avoid overlapping influence from any other strong source. Since the region around the power-plants is devoid of any other strong emission source, background emissions were assumed to be not more than 5 percentiles of the region’s emission.

### Anomaly metric

Anomaly was defined as per Eq. (2), to quantify the relative change between the weekly mean concentrations (or emissions) in 2020, $C_{2020, t}$, and the corresponding mean reference values, $C_{reference, t}$ for week-of-year $t$.

2 $$\begin{array}{*{20}c} {Anomaly = \frac{{C_{2020,t} - C_{reference,t} }}{{C_{reference,t} }} } \\ \end{array}$$

A high absolute anomaly implies a large deviation in $C_{2020}$ with respect to the reference $C_{reference}$ in the week $t$. During the lockdown weeks, anomaly was expected to be negative with large absolute values. Estimating anomaly as per the Eq. (2) also offers the benefit of removing systematic additive or multiplicative biases in satellite retrievals. Anomalies in OMI NO_2_ concentration in 2020 were calculated with respect to extrapolated concentration $C_{B}$ (defined in “Concentration change estimation”) as reference. Anomalies in top-down NOx emissions in 2020 were calculated with respect to the emission in 2019 as well as the mean BAU emission as reference.

## Results and discussion

### Spatial concentration change during lockdown

Spatial comparison of the TROPOMI NO_2_ column in 2020 with that in 2019 is shown as the mean anomaly for each lockdown-phase spatially in Fig. S7 and S8. It depicts a sharp decline in NO_2_ densities in urban areas with Phase 1 and a slow recovery in subsequent phases. Anomaly ($\pm 1\sigma$) during BAU was 0.01% (± 11.0%), reflecting that prior to lockdown overall densities were not different from the baseline, except at Rajiv Gandhi power-plant that showed a high positive anomaly (37.4%). During Phase 1 lockdown, overall densities had decreased substantially with a large negative mean anomaly (− 33.7% ± 12.1%), going as low as − 76.8% over central urban Delhi. Apart from urban areas relatively large negative anomalies were also found above power-plants and industries suggesting reduced emissions compared to 2019. Concurrently, compared to corresponding weeks in 2019, the zonal and meridional wind speeds during Phase 1 weeks were higher by 92% (mean 3.88 m/s) and 61% (mean − 2.49 m/s) respectively (see Section S1). During Phase 2, overall anomaly was reduced to − 2.1% ± 17.6% on account of a sharp spatial difference in anomaly between the rural western region (28.6%) falling in Rajasthan and the populated central (− 58.4%) and eastern regions (− 15.3%). A similar spatial dichotomy in anomalies was also found during Phase 3 with urban Delhi showing a 45.6% decline while rural areas showing a 36.5% increase. High positive anomalies observed in the western desert region is attributable to the small NO_2_ columns values wherein slight changes in concentrations appear as large anomalies. Overall anomaly during Phase 3 was − 7.2% ± 31.3%. During Phase 4, when most restrictions had been relaxed, NO_2_ concentration anomaly in Delhi reduced to − 37.7% from the − 76.8% during Phase 1. For the first time after the onset of Phase 1, concentration increased over the Panipat region, which has a power-plant and an oil refinery closely located to each other, showed a positive anomaly of 37.3%. Furthermore, Phase 4 coincided with the large-scale open wheat crop-residue burning in the northern regions of the domain, resulting in positive NO_2_ anomalies (Fig. S9). The overall anomaly for Phase 4 was − 4.5% ±  15.4%. During Phase 5, which saw no restrictions except in containment zones, concentration anomalies remained lower than that during BAU, suggesting that concentrations had still not recovered to the same levels as those in 2019 with overall anomaly as − 17.5% ± 12.3%. Lower anomalies may also have been partly contributed by unusual precipitation that took place during the latter half of June 2020^47^. The eastern-western spatial dichotomy was not apparent during Phase 5. Interestingly, negative anomalies were persistent over most of the urban region of Delhi and downwind regions of strong emitters such as power plants and refineries during all the phases while fluctuating in other regions.

### Concentration change during lockdown

However, the reduction in concentration cannot be conclusively calculated without adjusting for long-term trends. Reductions in concentrations were further analyzed by calculating anomaly with respect to the expected concentration. Furthermore, spatial differences in anomaly in the western and eastern portion of the domain suggests possible varying influence of wind speeds and direction in dispersing away the pollutants. This confounding by wind was addressed by estimating the impact on emissions (“Concentration change during lockdown”).

**Figure 2:**
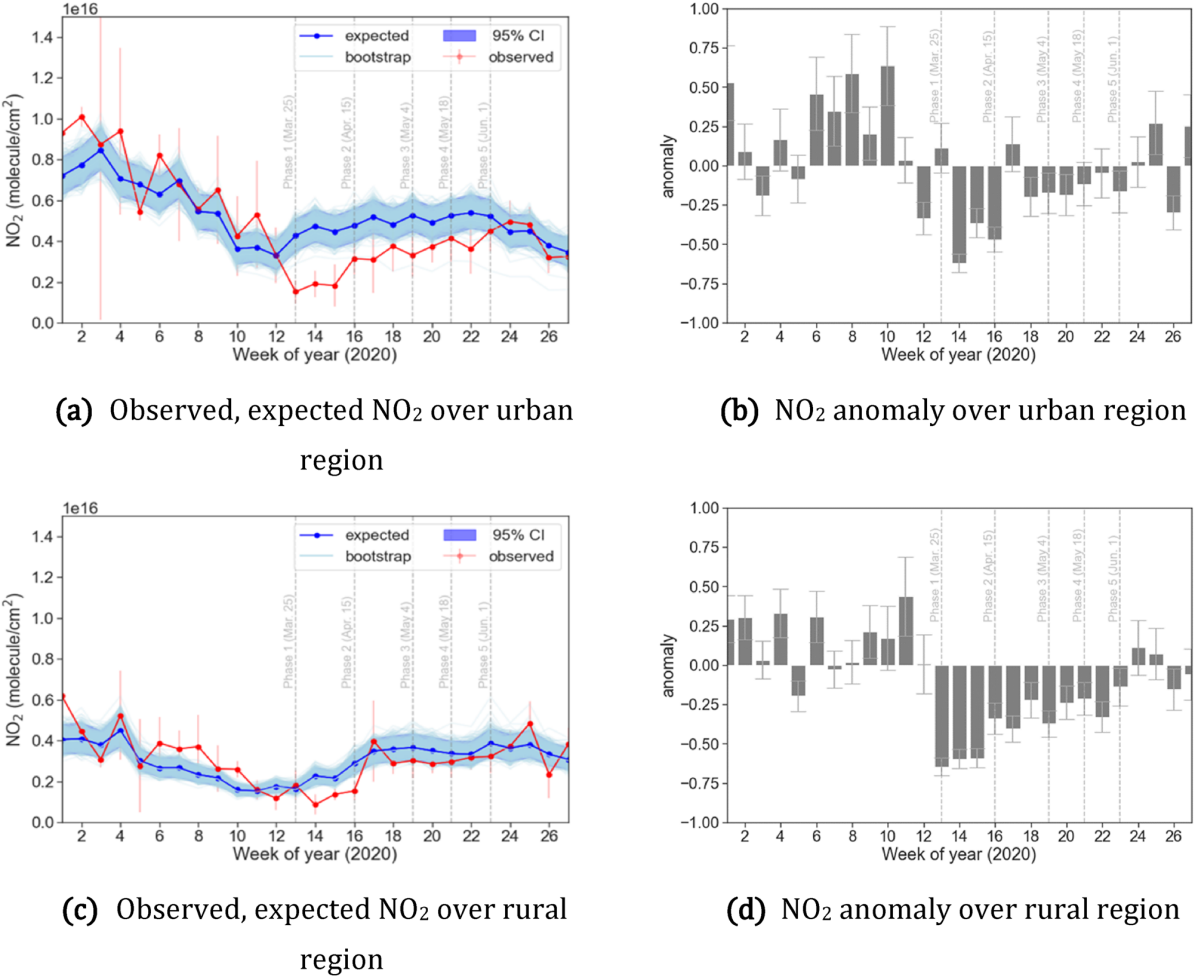
Weekly mean of observed ($C_{o}$) and expected ($C_{B}$) OMI NO_2_ concentration (left) and the anomaly between the observed and expected concentration (right) over Delhi (top) and rural Fatehabad (bottom). The anomaly range corresponds to 95% prediction intervals. Anomaly metric was calculated following Eq. (2).

Figure 2 shows the retrieved OMI NO_2_ concentration as well as the expected NO_2_ concentration over urban and the rural location and their corresponding anomalies for both the locations. Based on the STL decomposition, a small but significantly decreasing long-term linear-trend of NO_2_ from 2016 to 2019 was found over the urban Delhi (− 0.142 $\times 10^{15}$ year^−1^) as well as rural Fatehabad area. Seasonality of NO_2_ after STL decomposition shows high levels in the winter months (January and February) over Delhi and Fatehabad that gradually decreases with pre-monsoon summer. The seasonality corresponding to the photolysis rate, life-time and change in emissions. The photolysis rate of NO_2_ is proportionally related with actinic flux, which increases with decreasing solar zenith angle (as well as dependent on direct, diffused and reflected radiation governed by amount and type of the aerosols, absorbing gases, air molecules and the surface albedo^28^). A lower actinic flux during winter results in a lower NO_2_ photolysis frequency and OH radical production rate, thereby reducing the photochemical loss of NO_2_ and increasing its lifetime^36^. As the pre-monsoon dry summer with its high temperature starts approaching, actinic flux increases, thereby decreasing the lifetime of NOx and lowering NO_2_ concentration. Further with an onset of monsoon, wet deposition reduces the NO_2_.

As seen in Fig. 2a,c, the observed and expected NO_2_ concentrations during the BAU phase were similar, as evidenced by their relatively low mean NO_2_ anomaly over Delhi (15.7%). Similarly, a low mean anomaly was found for Fatehabad (20.3%) as shown in Fig. 2b,d. However, when the lockdown commenced, the urban NO_2_ showed a sharp deviation from the expected concentration. During the Phase 1 of lockdown, similar NO_2_ concentrations were observed in urban Delhi and rural Fatehabad. The largest negative anomaly over Delhi was observed during the first week of the Phase 1 as − 64.51% (− 56.9% to − 70.2%). However, for the rural Fatehabad a positive anomaly was observed during the first week of Phase 1 as 11.2% (− 4.7% to 31.8%) which during the second week of Phase 1 dipped to − 62.1% (− 68.0% to − 54.3%).

By comparing mean anomalies across lockdown- phase and locations (Table 1), it becomes clear that urban Delhi showed larger anomalies than rural Fatehabad and its reduced concentrations persisted for a longer duration than Fatehabad. Mean anomalies during the Phase 2 to Phase 4 in Delhi stayed between − 31 and − 27%, suggesting a slight recovery in emissions compared to Phase 1 reductions. During the same duration Fatehabad had lower mean anomalies ranging between – 8 and − 15%. This suggests that the impact of the lockdown on NO_2_ was not uniform in urban and rural areas. In urban areas, industrial and transportation activities did not resume to pre-COVID levels even during the unlock Phase 5 resulting in overall negative anomalies. However, in rural areas consistent smaller anomalies could be related to their permission to conduct agricultural activities (such as harvesting and transport) as usual as agricultural activities were excluded from the lockdown restrictions. Another reason could be that contribution of anthropogenic emissions from rural household biomass fuel burning as well as biogenic emission from agricultural soil^28^ kept on contributing to rural NO_2_ concentrations despite the lockdown (Table 1).

**Table 1:** Mean anomaly between the observed and expected NO_2_ concentrations for each lockdown Phase in 2020. The range corresponds to 95% prediction intervals.

Phase	Urban (Delhi)	Rural (Fatehabad)
BAU	15.7% (0.0% to 36.3%)	20.3% (5.6% to 44.0%)
Phase 1	− 61.0% (− 67.0% to − 53.0%)	− 33.5% (− 43.3% to 20.7%)
Phase 2	− 31.1% (− 42.0% to − 19.3%)	− 7.7% (− 22.0% to 10.8%)
Phase 3	− 30.4% (− 40.0% to − 17.4%)	− 15.0% (− 28.6% to 1.8%)
Phase 4	− 27.0% (− 37.0% to − 13.4%)	− 10.5% (− 25.0% to 6.6%)
Phase 5	− 3.4% (− 18.4% to 16.2%)	6.2% (10.4% to 27.3%)

### NOx emission change during lockdown

**Figure 3:**
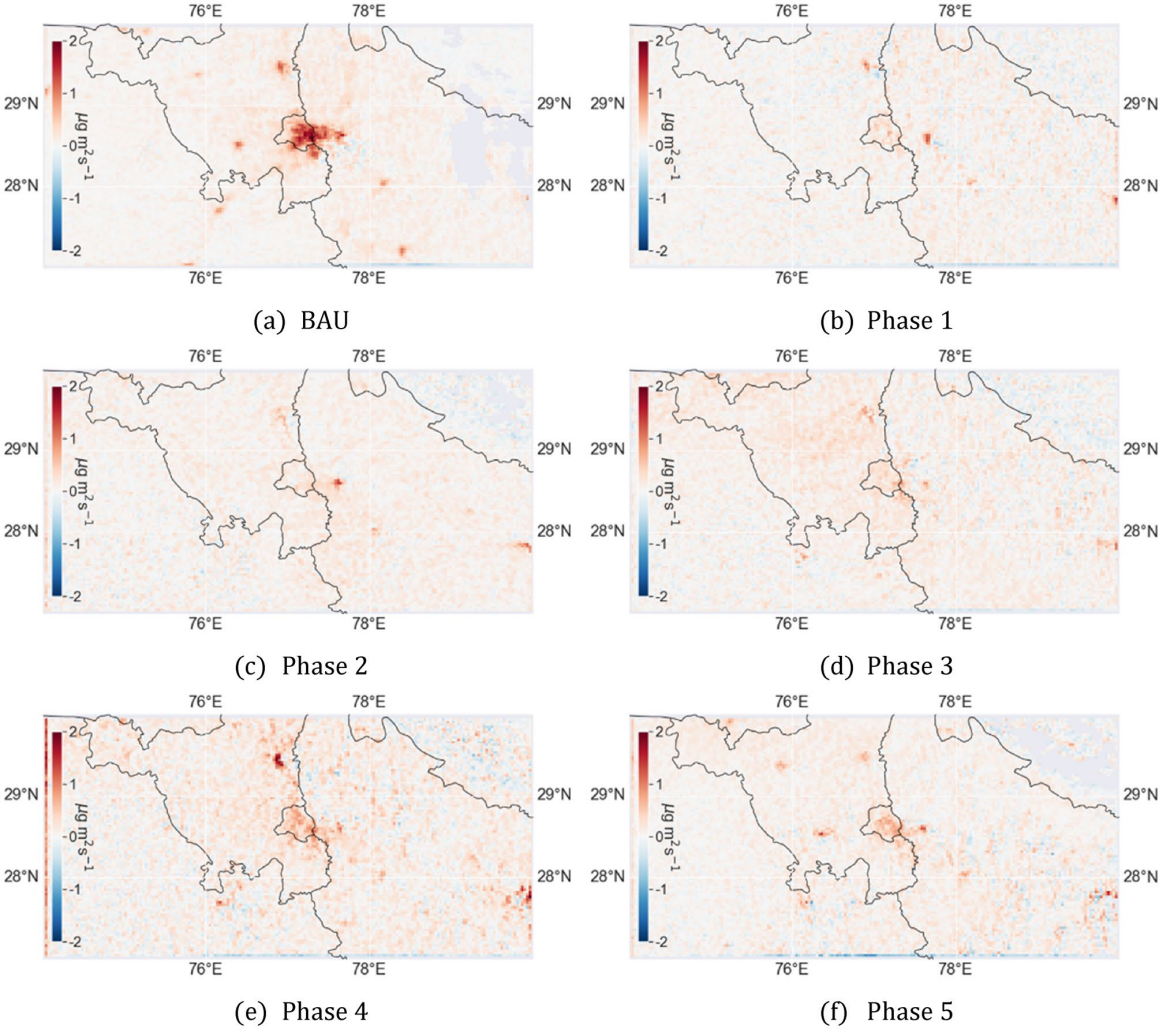
Mean top-down NOx emission in 2020 during (**a**) BAU (business-as-usual), and subsequent lockdown-phases (**b**–**f**). Figures generated using ‘Cartopy’ version 0.16 and ‘Rasterio’ version 1.2 modules of Python 3.6.

Top-down emissions during each phase is shown in Fig. 3. Compared to the concentration, smeared plumes are visible absent. Compared to BAU, substantial emission reduction was observed over urban Delhi as well as surrounding areas. As investigated earlier^48–50^ (also discussed in supplementary file), NOx emission in Delhi is mostly contributed by traffic and industries, amounting to 58% to 80% of total emissions. Apart from the urban areas, emissions from Harduaganj and Dadri power-plant as well as the oil refinery in Panipat were reduced substantially. Emission rates were similar in Phase 1, 2 and 3. During Phase 4, when most restrictions had been removed, emissions increased in areas within and surrounding Delhi as well as at the Panipat oil refinery. Such resumption of oil refining activities was also reported^51^. Also, several scattered weakly emitting point sources in northern Haryana, possibly corresponding to crop residue fires are visible. During the unlock Phase 5, emissions remained mostly similar to Phase 4, except at Panipat oil refinery where they had decreased compared to Phase 4 but higher than Phase 1.

**Figure 4:**
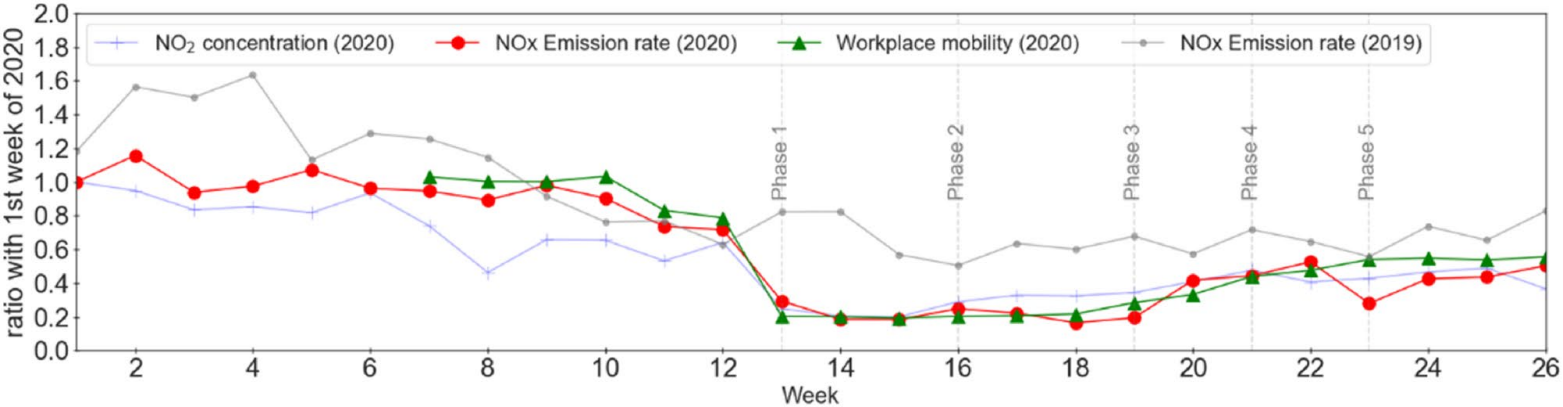
Mean NO_2_ concentration, change in workplace mobility and NOx emission in 2020 and 2019 standardized with respect to the first week of January 2020 (6 January–12 January, 2020) over Delhi.

A time-series comparison of top-down emission with activity reports for Delhi (Dadri power-plant and Harduaganj power-plant) is shown in Fig. 4 (Fig. S12). Interestingly, weekly emission from urban areas showed a slight decreasing trend between January and prior to lockdown in March (weeks 1 to 12). A similar was also observed for the emissions in 2019 due to the seasonal changes in primary NO_2_ emissions as need for biomass fuel burning reduce. It is possible that relatively higher emission estimation in January was due to burning of biomass for heating^28^. It is also possible that emission is slightly underestimated in March due to the assumption of a higher than actual $\tau$, which may be unlikely due to an increased photolysis rate during March. Over these three locations (urban Delhi, Harduaganj power-plant and Dadri power-plant), correlation between the reported activity and top-down emission is higher than the correlation between the activity and concentration (Table S2). This confirms that changes in top-down emissions assess the lockdown impacts better than the concentration since the emission overcome confounding from wind-field related meteorological effects. However, for power-plants, the difference between activity-concentration correlation and the activity-emission correlation is small suggesting that over strong point sources lockdown impacts can be equally informed by changes in either emissions or concentrations. A high correlation between the emission and the mobility activity over the urban area (0.96) confirms that most NO_2_ emissions are related with people’s movement activities from transportation as also suggested in previous bottom-up emission inventories studies.

Emission anomalies were calculated by comparing the emission during lockdown-phases with the mean emission in the three weeks prior to the lockdown. Emission during Phase 1 decreased by 72.2% over the urban area which by Phase 5 had recovered to 49.5% decrease. At the same time, compared to the baseline, workplace mobility had decreased by average 80.6% in the initial Phase 1 and by 45.5% in Phase 5. At the Harduaganj and Dadri power-plants, lower demands led to a power production decrease by 20% and 55% respectively. At the corresponding locations mean emission decreased by 53.4% and 48.5% during Phase 1 and Phase 2 compared to BAU.

Emission anomalies in 2020 were also calculated with respect to the corresponding emissions of 2019 as reference. Emissions anomalies so calculated are visualized spatially for each phase in Fig. S3 and summarized for Delhi in Table 2. As transportation and factories came to standstill during the Phase1, for Delhi about 72% NOx emissions can be attributed solely to the traffic and factories.

**Table 2:** Mean top-down NOx emission (± standard deviation) in 2020 and its anomaly with respect to 2019 emissions over a 60 × 60 km^2^ region around central Delhi (77.21 E, 28.60 N).

Phase	Mean emission (kg/s)	Mean anomaly compared to 2019
BAU	2.78 ± 0.43	− 17.3%
Phase 1	0.61 ± 0.13	− 72.0%
Phase 2	0.62 ± 0.09	− 63.2%
Phase 3	0.85 ± 0.29	− 50.3%
Phase 4	1.30 ± 0.72	− 29.4%
Phase 5	1.05 ± 0.18	− 40.6%

### Uncertainty in satellite retrieved tropospheric NO_2_ and top-down NOx emissions

Tropospheric NO_2_ retrievals suffer uncertainty in slant column density (due to measurement noise and spectral fitting errors), stratospheric slant column (due to error in separating stratospheric and tropospheric NO_2_) and tropospheric AMF (due to model parameter errors such as assumed profile shape^52^). The trace gas vertical profile is needed to derive VCD by separating AMF from SCD. Column uncertainty due to AMF is about 30%^52^. If the satellite retrieval assumed NO_2_ height profile has a smaller aerosol fraction close to surface compared to the true profile, then tropospheric AMF will be overestimated and correspondingly the retrieved tropospheric NO_2_ VCD will be underestimated^52^. Shaiganfar et al. compared OMI NO_2_ VCD with MAX-DOAS observations over Delhi and found that OMI retrievals underestimate high concentrations. Over highly polluted regions tropospheric NO_2_ VCDs are partially underestimated due to shielding of emitted NO_2_ by aerosols^42^. The random errors are reduced by temporal and spatial averaging, while a major part of systematic errors is expected to cancel out through the difference and ratio in the defined anomaly metric.

Estimation of total emission is complicated mainly by the variation in wind field and chemical transformation^26,53^. As long as the wind has constant speed and direction and these parameters are known with low uncertainty, the steady-state assumption can be applied. However slow winds which change directions with time and space complicate emission estimation as such scenarios have high uncertainty (relative to wind speed) and the sudden change in wind direction breaks the steady-state assumption. However, the error due to non-stationary state is smaller near point sources, which can be further diminished by taking a multi-temporal mean. Another problem is that while the wind speeds and directions change with altitude, the vertical profile of the trace gas itself is not well-known. For example, the near-surface emissions (from vehicles) and chimney-stack emissions are injected at different altitudes and their dispersion is subjected to different wind speeds. Chemical reaction rates may also add uncertainties in the assumed lifetime and the constant Leighton ratio. As stated earlier NO_2_/NOx ratio is lower when close to the freshly emitting source in space and time, which increases in aged plumes after chemical conversion^44^. Near strong emission sources emitted NO may not be quickly converted to NO_2_ if the NO mixing ratios locally exceed those of ozone. The NO/NO_2_ steady state is completely achieved only after ambient air has mixed with the emitted plume^42^. Turbulence in wind speed may enhance mixing and conversion of NO to NO_2_ near the source or it may disperse NO downwind before it gets converted to NO_2_^44^. We assumed a constant ratio of NO/NO_2_, neglecting possible changes by day of the week.

## Conclusions

The natural experiment induced by COVID-19 lockdown was used to assess the impact of lockdown on NOx concentration and anthropogenic emissions in a region in Northern India around Delhi. To estimate the decline in NO_2_ during the lockdown, seasonality and trend decomposition was used to calculate the expected NO_2_ in 2020 by extrapolating it from previous years. Compared to the expected concentrations, NO_2_ concentration in urban areas reduced by 60% in Phase 1 of lockdown to 3% in Phase 5 of lockdown. In contrast, the rural areas exhibited much smaller and short-lived reductions suggesting a lower impact of the movement restriction policies. To estimate the detailed impact of the lockdown top-down emissions were estimated spatially using steady state continuity equation by considering the transport of pollutants. The calculations suggest compared to the business-as-usual phase of 2020, emission over the urban area and power-plants decreased by 72.2% and 53.4%, respectively during the Phase 1 of lockdown. By Phase 5, emissions over urban areas had recovered, showing a decrease of 49.5% compared to business-as-usual phase of 2020. Higher correlation of activity reports with emission compared to concentrations, suggested that top-down emissions are representative for estimating the impact of lockdown specially over urban areas. This analysis can be extended to highlight the varying impacts of diverse policy regulations on NOx emission sources in different locations and better understand the sources contributing to air pollution in each location.

## Supplementary Information

Supplementary Information.

## Data Availability

TROPOMI was processed and downloaded from Google Earth Engine, OMI was downloaded from NASA’s Giovanni platform and meteorological data obtained from NCAR’s Research Data Archive. Original code was developed by the authors for top-down emission estimation in Python language and it is available from the corresponding author upon request.

## References

[1] Bonaccorsi G (2020). Economic and social consequences of human mobility restrictions under COVID-19. Proc. Natl. Acad. Sci. U. S. A..

[2] McKibbin W, Fernando R (2020). The Global Macroeconomic Impacts of COVID-19: Seven Scenarios. Asian Econ. Pap..

[3] Dhaka SK (2020). PM(25) diminution and haze events over Delhi during the COVID-19 lockdown period: An interplay between the baseline pollution and meteorology. Sci. Rep..

[4] Diamond MS, Wood R (2020). Limited regional aerosol and cloud microphysical changes despite unprecedented decline in nitrogen oxide pollution during the February 2020 COVID-19 shutdown in China. Geophys. Res. Lett..

[5] Chauhan A, Singh RP (2020). Decline in PM25 concentrations over major cities around the world associated with COVID-19. Environ. Res..

[6] Chen R (2012). Associations between short-term exposure to nitrogen dioxide and mortality in 17 Chinese cities: The China Air Pollution and Health Effects Study (CAPES). Environ. Int..

[7] Zhao, Y. *et al.* [ASAP] Substantial Changes in Nitrate Oxide and Ozone after Excluding Meteorological Impacts during the COVID-19 Outbreak in Mainland China. *Environ. Sci. Technol. Lett.* acs.estlett.0c00304 (2020). doi:10.1021/acs.estlett.0c00304 10.1021/acs.estlett.0c00304 37566301

[8] Shi X, Brasseur GP (2020). The Response in Air Quality to the Reduction of Chinese Economic Activities During the COVID-19 Outbreak. Geophys. Res. Lett..

[9] Le T (2020). Unexpected air pollution with marked emission reductions during the COVID-19 outbreak in China. Science.

[10] Bauwens M (2020). Impact of coronavirus outbreak on NO2 Pollution Assessed Using TROPOMI and OMI observations. Geophys. Res. Lett..

[11] Singh V (2020). Diurnal and temporal changes in air pollution during COVID-19 strict lockdown over different regions of India. Environ. Pollut..

[12] Singh RP, Chauhan A (2020). Impact of lockdown on air quality in India during COVID-19 pandemic. Air Qual. Atmos. Health.

[13] Kotnala G, Mandal TK, Sharma SK, Kotnala RK (2020). Emergence of blue sky over delhi due to coronavirus disease (COVID-19) lockdown implications. Aerosol Sci. Eng..

[14] Mahato S, Pal S, Ghosh KG (2020). Effect of lockdown amid COVID-19 pandemic on air quality of the megacity Delhi, India. Sci. Total Environ..

[15] Kumar P (2020). Temporary reduction in fine particulate matter due to ‘anthropogenic emissions switch-off’ during COVID-19 lockdown in Indian cities. Sustain. Cities Soc..

[16] Sharma S (2020). Effect of restricted emissions during COVID-19 on air quality in India. Sci. Total Environ..

[17] Lamsal LN (2015). U.S. NO2 trends (2005–2013): EPA Air Quality System (AQS) data versus improved observations from the Ozone Monitoring Instrument (OMI). Atmos. Environ..

[18] Vlemmix T (2015). MAX-DOAS tropospheric nitrogen dioxide column measurements compared with the Lotos-Euros air quality model. Atmos. Chem. Phys..

[19] Ding J (2020). NOx emissions reduction and rebound in China due to the COVID-19 crisis. Geophys. Res. Lett..

[20] Miyazaki K (2020). Air quality response in China linked to the 2019 novel coronavirus (COVID-19) lockdown. Geophys. Res. Lett..

[21] Zhang R (2020). NOx emission reduction and recovery during COVID-19 in East China. Atmosphere.

[22] Shi Z (2021). Abrupt but smaller than expected changes in surface air quality attributable to COVID-19 lockdowns. Sci. Adv..

[23] Goldberg DL (2020). Disentangling the impact of the COVID-19 lockdowns on urban NO2 from natural variability. Geophys. Res. Lett..

[24] Martin RV (2003). Global inventory of nitrogen oxide emissions constrained by space-based observations of NO 2 columns. J. Geophys. Res..

[25] Lorente A (2019). Quantification of nitrogen oxides emissions from build-up of pollution over Paris with TROPOMI. Sci. Rep..

[26] Beirle S (2019). Pinpointing nitrogen oxide emissions from space. Sci. Adv..

[27] Misra P (2020). Mapping brick kilns to support environmental impact studies around delhi using sentinel-2. ISPRS Int. J. Geo-Inf..

[28] Ghude SD, Fadnavis S, Beig G, Polade SD, der Van ARJ (2008). Detection of surface emission hot spots, trends, and seasonal cycle from satellite-retrieved NO2 over India. J. Geophys. Res. Atmos..

[29] Ghude SD (2013). Application of satellite observations for identifying regions of dominant sources of nitrogen oxides over the Indian subcontinent. J. Geophys. Res. Atmos..

[30] Levelt PF (2006). The ozone monitoring instrument. IEEE Trans. Geosci. Remote Sens..

[31] Griffin D (2019). High-resolution mapping of nitrogen dioxide With TROPOMI: First results and validation over the Canadian oil sands. Geophys. Res. Lett..

[32] Boersma KF (2011). An improved tropospheric NO2 column retrieval algorithm for the Ozone Monitoring Instrument. Atmos. Meas. Tech..

[33] Nitta, K., Misra, P. & Hayashida, S. Intercomparison of TROPOMI and OMI Tropospheric Nitrogen Dioxide over South Asia (submitted). *Remote Sens.*

[34] Acker J, Soebiyanto R, Kiang R, Kempler S (2014). Use of the NASA giovanni data system for geospatial public health research: Example of weather-influenza connection. ISPRS Int. J. Geo-Inf..

[35] Gorelick N (2017). Google Earth Engine: Planetary-scale geospatial analysis for everyone. Remote Sens. Environ..

[36] Beirle S, Platt U, Wenig M, Wagner T (2003). Weekly cycle of NO2 by GOME measurements: A signature of anthropogenic sources. Atmos. Chem. Phys..

[37] NCEP. National Centers for Environmental Prediction/National Weather Service/NOAA/U.S. Department of Commerce. 2000, updated daily. *NCEP FNL Operational Model Global Tropospheric Analyses, continuing from July 1999*. 10.5065/D6M043C6 (2000).

[38] Le Quéré C (2020). Temporary reduction in daily global CO2 emissions during the COVID-19 forced confinement. Nat. Clim. Change.

[39] POSOCO. *Daily Regional Power Supply Position*. https://nrldc.in/reports/daily-reports/daily-regional-power-supply-position/. (Accessed 21 May 2020).

[40] Google. *COVID-19 Community Mobility Reports*. https://www.google.com/covid19/mobility/. (Accessed 21 May 2020).

[41] Cleveland RB, Cleveland WS, McRae JE, Terpenning I (1990). STL: A seasonal-trend decomposition procedure based on Loess. J. Off. Stat..

[42] Shaiganfar R (2011). Estimation of NO x emissions from Delhi using Car MAX-DOAS observations and comparison with OMI satellite data. Atmos. Chem. Phys..

[43] Seinfeld JH (1989). Urban air pollution: State of the science. Science.

[44] Kimbrough S, Chris Owen R, Snyder M, Richmond-Bryant J (2017). NO to NO2 conversion rate analysis and implications for dispersion model chemistry methods using Las Vegas, Nevada near-road field measurements. Atmos. Environ..

[45] Chatani S (2018). Overview of model inter-comparison in Japan’s study for reference air quality modeling (J-STREAM). Atmosphere.

[46] Beirle S, Boersma KF, Platt U, Lawrence MG, Wagner T (2011). Megacity emissions and lifetimes of nitrogen oxides probed from space. Science.

[47] New Delhi, India Weather History | Weather Underground. Available at: https://www.wunderground.com/history/monthly/in/new-delhi/VIDD/date/2020-6. (Accessed: 18th September 2020)

[48] Sahu SK (2015). High Resolution Emission Inventory of NOx and CO for Mega City Delhi, India. Aerosol Air Qual. Res..

[49] Sindhwani R, Goyal P, Kumar S, Kumar A (2015). Anthropogenic emission inventory of criteria air pollutants of an urban agglomeration: National capital region (NCR), Delhi. Aerosol Air Qual. Res..

[50] Guttikunda SK, Calori G (2013). A GIS based emissions inventory at 1 km x 1 km spatial resolution for air pollution analysis in Delhi, India. Atmos. Environ..

[51] Indian Oil’s refineries to operate at 80% capacity. *The Hindu* (2020). https://www.thehindu.com/business/indian-oils-refineries-to-operate-at-80-capacity/article31560534.ece. (Accessed: 17th September 2020)

[52] Boersma KF, Eskes HJ, Brinksma EJ (2004). Error analysis for tropospheric NO2 retrieval from space. J. Geophys. Res. Atmos..

[53] Ibrahim O (2010). Car MAX-DOAS measurements around entire cities: Quantification of NOx emissions from the cities of Mannheim and Ludwigshafen (Germany). Atmos. Meas. Tech..

