# Supplementary material for: Nitrogen oxides concentration and emission change detection during COVID-19 restrictions in North India
Source: Sci Rep. 2021 May 7;11:9800. doi: 10.1038/s41598-021-87673-2 (PMC8105320; doi:10.1038/s41598-021-87673-2)
Supplement: Supplementary file 1 — Supplementary Information. [file 41598_2021_87673_MOESM1_ESM.pdf]

Supplementary File

Nitrogen Oxides Concentration and Emission change detection during COVID-19 restrictions in North India

Prakhar Misra[1*], Masayuki Takigawa[2], Pradeep Khatri[3], S.K. Dhaka[4], A.P. Dimri[5], Kazuyo Yamaji[6], Mizuo Kajino[7], Wataru Takeuchi[8], Ryoichi Imasu[9], Kaho Nitta[10], Prabir K. Patra[2], Sachiko Hayashida[1,10]

[1] Research Institute for Humanity and Nature, Kyoto, Japan; mprakhar@chikyu.ac.jp
[2] Japan Agency for Marine-Earth Science and Technology, Yokohama, Japan
[3] Graduate School of Science, Tohoku University, Sendai, Japan
[4] Radio and Atmospheric Physics Lab, Rajdhani College, University of Delhi, New Delhi, India
[5] School of Environmental Sciences, Jawaharlal Nehru University, New Delhi, India
[6] Kobe University, Kobe, Japan
[7] Meteorological Research Institute, Japan Meteorological Agency, Tsukuba, Japan
[8] Institute of Industrial Science, The University of Tokyo, Tokyo, Japan
[9] Atmosphere and Ocean Research Institute, The University of Tokyo, Chiba, Japan
[10] Faculty of Science, Nara Women's University, Nara, Japan
* Correspondence: mprakhar@chikyu.ac.jp; Tel.: (+81 70-4813-2297)

This file includes a further discussion on:

S1. Meteorological variability between 2020 and 2019

S2. Trend of OMI retrieved NO_2

S3. Comparison between OMI and TROPOMI tropospheric NO_2

S4. Spatial change in TROPOMI tropospheric NO2

S5. Biomass-fire

S6. Top-down NOx emission estimation

S7. Emission uncertainties

Figure S1 to S13

Table S1 to S3

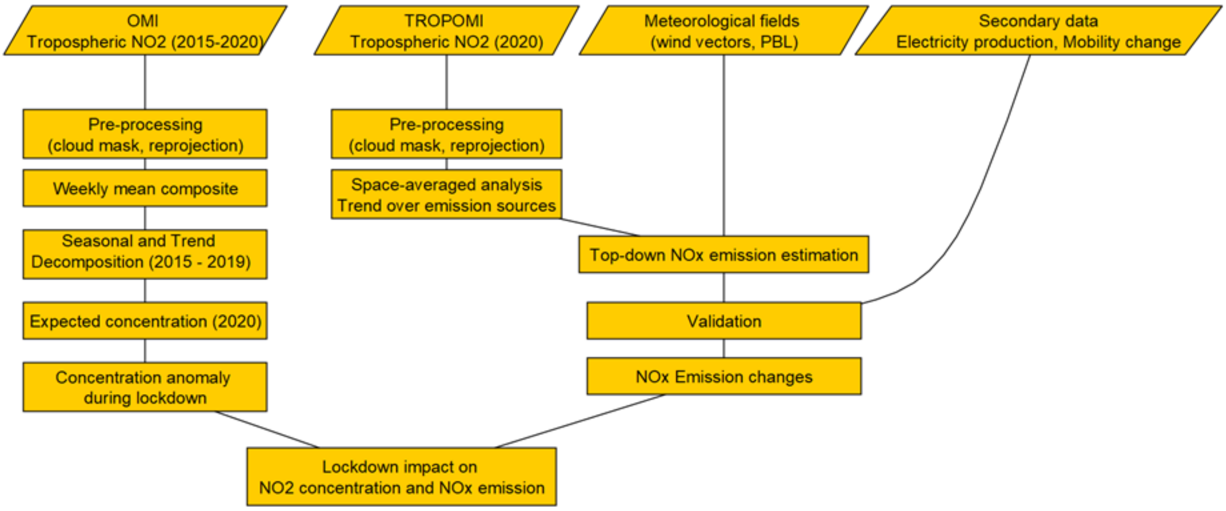

Figure S1. Analysis flowchart of the study. Figure generated using 'yEd Graph Editor' version 3.19.

Table S1. A brief timeline of the policy restrictions pertaining to COVID-19 in India in 2020 until June 30, 2020. Phase 1-4 are "lockdown" phases, while Phase 5 is "unlock" phase.

Restrictions	Applicable dates	Policy measures
BAU	January 1 to March 22	Business-as-usual, no restriction with respect to COVID-19
Curfew	March 22	Nationwide curfew
Phase 1	March 25 - April 14 (21 days)	Starting during week 13, all non-essential service and factories were suspended (Ministry of Home Affairs 2020a).
Phase 2	April 15 – May 3 (19 days)	Starting during week 16, agricultural businesses, public work program, cargo transportation and banks were opened after April 20 (Ministry of Home Affairs 2020a).
Phase 3	May 4 – May 17 (14 days)	Starting during week 19, zonification of lockdown areas was performed as "red", indicating the presence of infection hotspots, "orange" indicating some infection, and "green" with no infections. Red zones were further demarcated as buffer and containment zones. Construction activities and traffic movement was relaxed for green and orange zone while red zones remained in lockdown (Ministry of Home Affairs 2020a).
Phase 4	May 18 – May 31 (14 days)	Starting during week 21, except venues of mass gatherings such as schools, colleges, shopping malls, air travel and others all other activities were permitted (Ministry of Home Affairs 2020a).
Phase 5	June 1 – June 30 (30 days)	Starting from week 23, shopping malls, religious places, hotels and restaurants were permitted to reopen. Only containment zones remained in lockdown (Ministry of Home Affairs 2020b).

S1. *Meteorological variability between 2020 and 2019*

One of the reasons why comparison of concentration between 2020 and 2019 alone could be misleading is because interannual meteorological variability plays an important role in determining NO_2 concentration, particularly wind speeds as they regulate horizontal advection[1]. A 10% difference in wind speeds around 6.5m/s can cause a difference in NO2 column concentration by 10%, especially in winter season[1]. We chose 80m for the analysis because 80m is a typical height of the "surface boundary layer" in the planetary boundary layer. As the temperature profile shows diurnal cycle in a layer below 200m or 300m height, and 80m height is just the central height in the layer, where wind is not so strongly affected by the surface.

Mean weekly trend of zonal (u)and meridional wind speed (v) at 80m height, well as the planet boundary layer height (*pblh*), as it regulates the intra-day vertical diffusion, is shown in Figure S2. Compared to their values in 2019, during the Phase 1 (starting week 13), u was higher by 92% (mean 3.88 m/s), v was higher by 61% (mean -2.49 m/s). During weeks 17 to 19, u was slower by 70% (mean 1.3m/s). Concurrently, compared to 2019, *pblh* was about 28% higher in the eight weeks preceding the lockdown and lower by about 13% in the eight weeks beginning with the lockdown. The variation in *pblh* is more important for surface observation than the satellite retrievals which are column based.

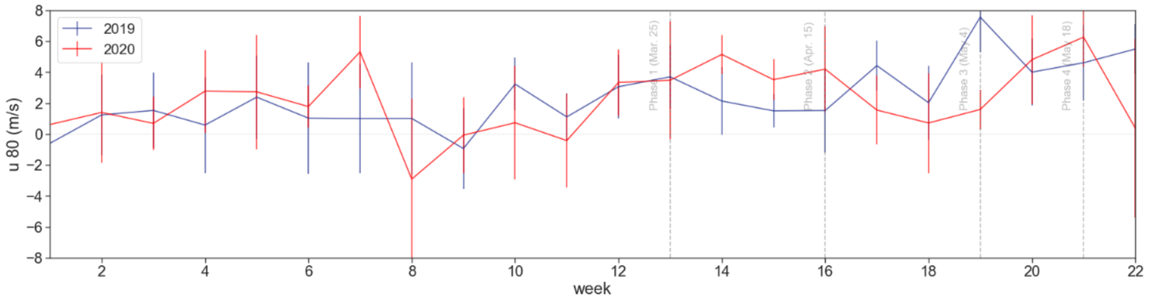

(a) zonal wind speed

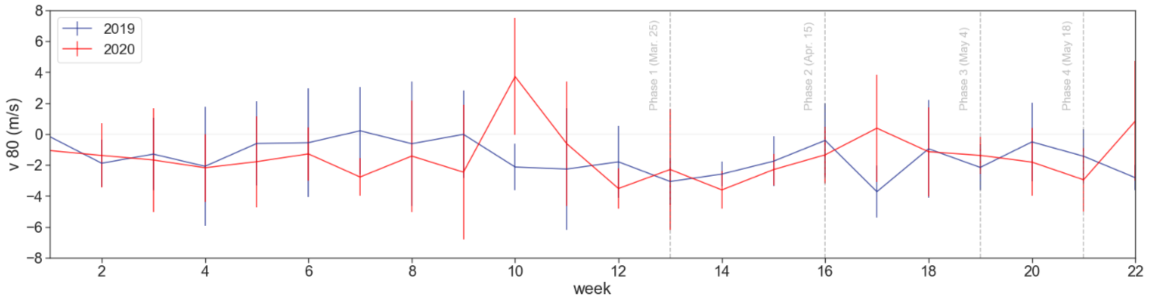

(b) meridional wind speed

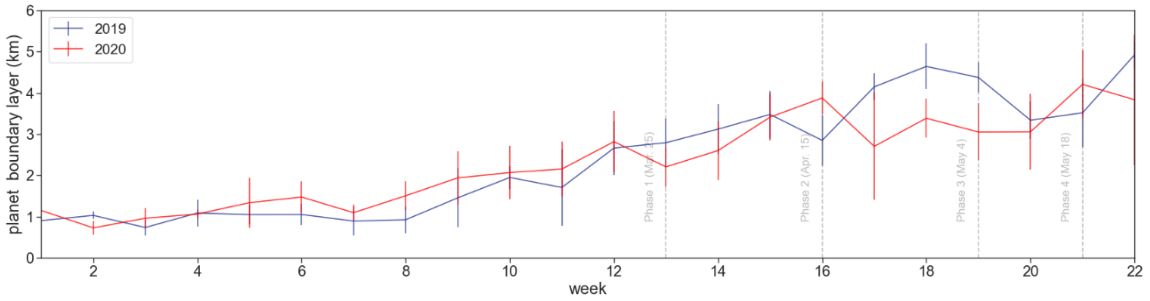

(c) planetary boundary layer height

Figure S2. Weekly mean (a) zonal wind speed, (b) meridional wind speed at 80-meter altitude, and (c) planetary boundary layer height of 2020 and 2019 over the Delhi, highlighting the yearly difference in the key meteorological variables that affect advection of NO_2.

S2. *Trend of OMI retrieved NO_2*

Although OMI was designed for daily retrievals, since June 2007 it has suffered a so-called 'row-anomaly' where its radiance measurements in some field of views have been affected by obstruction[2]. As a consequence of "row anomaly", OMI is missing data for almost half of its observation range, so the world-wide coverage is currently achieved in about two-days [3]. In this study, we used the product, 'OMNO2d' version 3, from NASA's Giovanni portal wherein the row anomaly flagged pixel and image with more than 30% cloud-fractions have been filtered already. To further analyze the impact of such filtering, we checked the weekly frequency of valid daily retrievals over a sample location, (urban Delhi: 76.875°E, 28.375°N - 77.625°E, 28.875°N) for 6 years (2015 to 2020) as shown in **Error! Reference source not found.** (a). Retrievals were the lowest in January (weeks 1 to 4, median: 3 /week) followed by February (weeks 4 to 8, median: 4/week) and December (weeks 48 to 52, median: 4/week). For all other months the median weekly frequency of daily retrievals was higher than 4. A frequency 5/week was judged to sufficiently represent the weekly concentration. Based on the lockdown phases. we were interested mainly the retrievals made during the first six months of a year. Coincidently, the lockdown was initiated from week 13 to 27, where the median weekly retrieval count is at least 5/week and the inferences made from OMI retrievals in these weeks could be used confidently. However inferences made for preceding weeks, especially those of January (weeks 1 to 4) could have higher uncertainties.

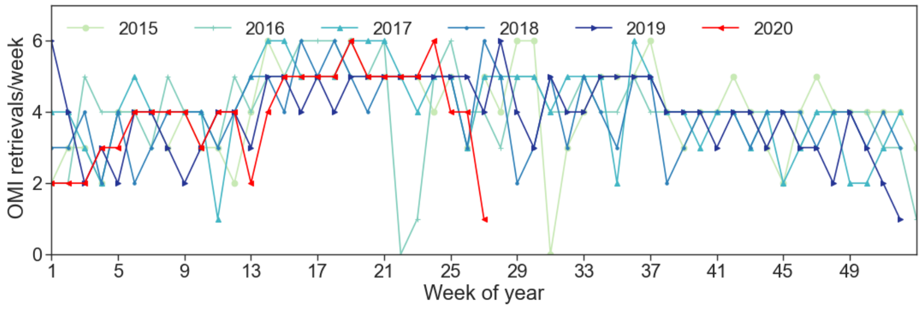

Figure S3. Number of valid daily retrieval per week from OMI sensor.

The weekly mean NO_2 concentrations at urban (Delhi) and rural (Fatehabad) for the weeks 1 to 27 are shown in Figure S4 (a) and (b). The seasonal is visually trend is similar to what has been reported earlier based on ground studies[4]. The relatively large standard deviation in the weeks (weeks 1 to 8) could be due to low number of retrievals in those weeks.

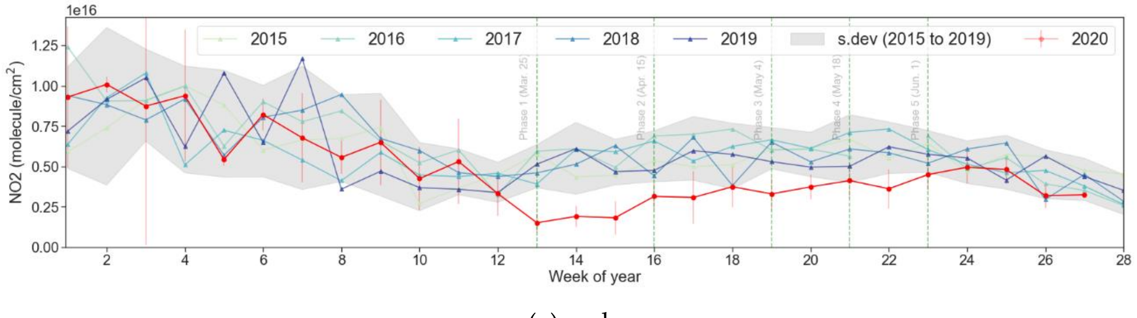

(a) urban

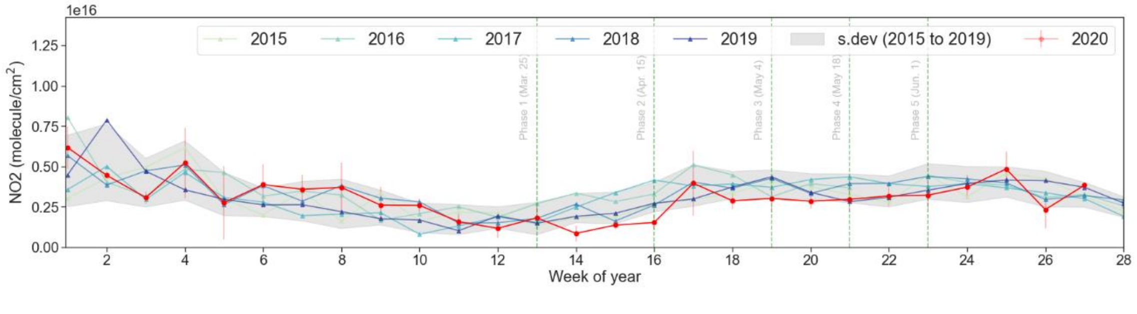

(b) rural

Figure S4. Trend and standard deviation of OMI retrieved weekly mean NO_2 column concentrations over (a) New Delhi and (b) rural background location.

S3. *Comparison between OMI and TROPOMI tropospheric NO_2*

It has been pointed out earlier that OMI retrievals are systematically lower than the ground-based Multi-AXis Differential Optical Absorption Spectroscopy (MAX-DOAS) column measurements in strongly polluted areas and vice versa in low concentration areas[5]. TROPOMI retrievals have a tendency to slightly overestimate MAX-DOAS columns by 7% over clean regions (below $2 \times 10^{15}\text{molec/cm}^2$) and underestimate by 36% over polluted regions (between $3 - 14 \times 10^{15}\text{molec/cm}^2$), wherein low NO_2 column values are better reproduced than high NO2 column values[6]. The bias is thus within the mission requirement of 50%. Global comparison between OMI and TROPOMI has revealed that due to the difference in pixel resolution, representativeness remains a major issue: OMI's larger ground pixels, particularly near the swath-edges different amounts of NO_2 and of cloud cover[6]. Over polluted areas TROPOMI is lower than OMI's, with differences varying from a few to -40% (in the winter) while in very clean regions TROPOMI has higher values by about 20%[6]. Over India, the difference between OMI and TROPOMI, the bias of TROPOMI is $-0.2 \pm 0.8 \times 10^{15}(1\sigma)$ molec/cm^2, which is $-6\% \pm 21\%$ in relative value[7].

A comparison between the two products was performed over the polluted urban region (Delhi, mean annual TROPMI concentration in 2019: $5.7 \times 10^{15}\text{molec/cm}^2$) and the clean rural location (Fatehabad, mean annual TROPOMI concentration in 2019: $2.1 \times 10^{15}\text{molec/cm}^2$) for the period Jan. 2019 to Jun. 2020. The difference between the weekly mean of TROPOMI and OMI columns in 2019 was analyzed. Both the products showed a similar seasonality at urban and rural locations (Figure S5). Out of the 52 weeks of 2019, over urban (rural) areas 32 (36) weeks had at least 4 daily retrievals devoid of cloud or row-anomaly. Over the urban area, mean weekly difference between TROPOMI and OMI was $0.3 \pm 1.3 \times 10^{15}\text{molec/cm}^2$ $(6.4\% \pm 23.1\%)$, and was biased high during high pollution (regimes greater than $4.8 \times 10^{15}\text{molec/cm}^2$), which often occurred during the winter months. Additionally, the difference and its deviation was lower ($0.2 \pm 0.7 \times 10^{15}\text{molec/cm}^2$) in the weeks when at least 4 daily valid retrievals existed. Over the rural region, TROPOMI was consistently lower than OMI by a mean value of $0.9 \pm 0.8 \times 10^{15}\text{molec/cm}^2$ $(39.9\% \pm 39.1\%)$. The difference between the two products was smaller ($0.7 \times 10^{15}\text{molec/cm}^2$) in the weeks with at least 4 daily retrievals.

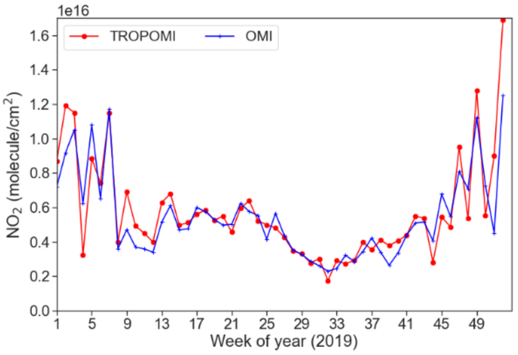

S4. urban

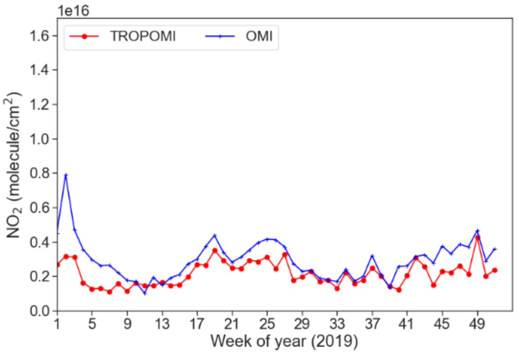

S5. rural

Figure S5. Weekly mean 2019 NO2 concentrations from TROPOMI and OMI sensors over an urban and rural region.

Furthermore, the relative difference in the concentrations between 2020 and 2019 from TROPOMI and OMI was also analyzed over the two locations (shown in Figure S6). The seasonal trend of the relative difference shows a greater consistency over the urban area compared to the rural area. Over the urban area, the mean relative difference (anomaly) in the pre-lockdown weeks (week

1 to week 12) of TROPOMI and OMI was -0.5×10^{15}molec/cm^2 (6.5%) and 0.1×10^{15}molec/cm^2(13.5%) respectively, while in the lockdown weeks (week 13 to 27) was -2.2×10^{15}molec/cm^2 (-38.1%) and -1.9×10^{15}molec/cm^2(-34.9%) respectively. Over the rural area, the mean relative difference (anomaly) of TROPOMI and OMI in the pre-lockdown weeks was 0.2×10^{15}molec/cm^2(19.9%) and 0.3×10^{15}molec/cm^2(22.3%) respectively, while in the lockdown weeks it stood at -0.4×10^{15}molec/cm^2(-15.9%) and -0.3×10^{15}molec/cm^2(-11.9%) This shows that although the difference of TROPOMI and OMI with regards to their relative difference or the anomaly was higher during the pre-lockdown months, the difference was lower during the lockdown period. It suggests that despite the underestimation in TROPOMI columns compared to the OMI columns, the calculated anomaly from both the sensors is quite similar and specially in lockdown weeks it is within 5% of each other.

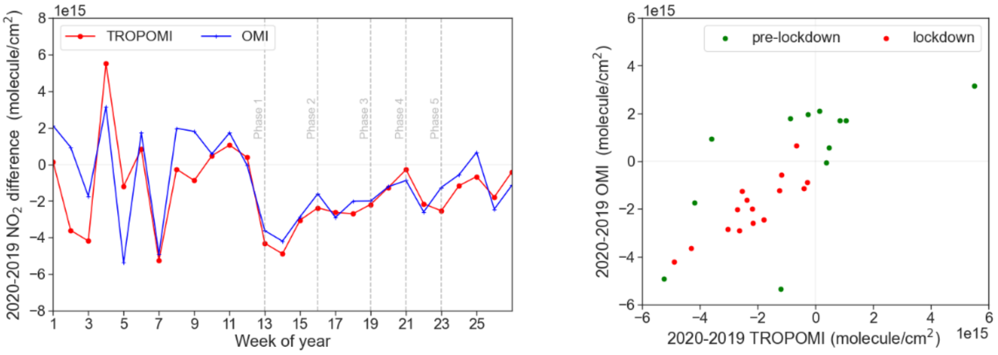

(a) Urban region

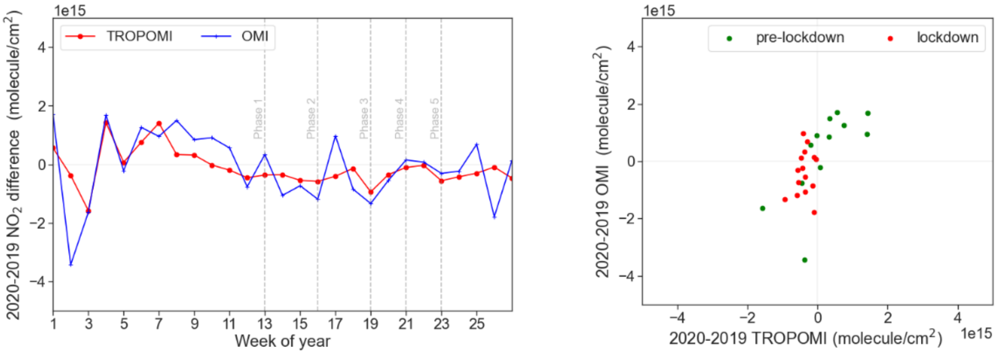

(b) Rural region

Figure S6. Weekly relative difference of 2020 and 2019 NO_2 concentration trend and scatter-plot from the TROPOMI and OMI sensor over an (a) urban and (b) rural region. Both sensors follow a similar trend of the relative difference. The difference of OMI and TROPOMI relative difference is within 5% of each other for weeks under lockdown (weeks from 13 to 27).

S4. *Spatial change in TROPOMI tropospheric NO_2*

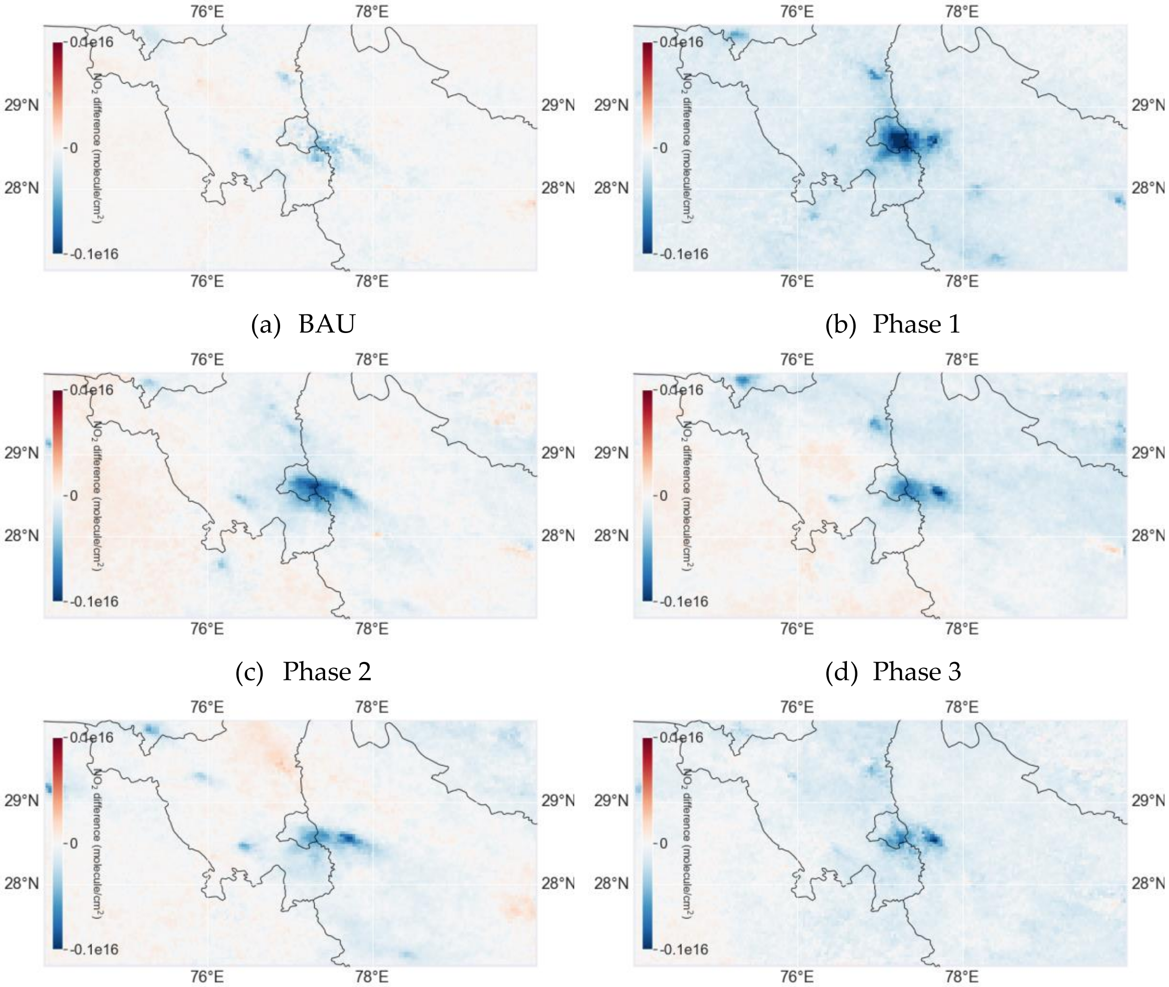

(a) BAU (b) Phase 1

(c) Phase 2 (d) Phase 3

(e) Phase 4 (f) Phase 5

Figure S7. Lockdown phase-wise mean relative difference of TROPOMI NO_2 columns in 2020 with respect to 2019. Pre-lockdown business-as-usual phase is denoted as BAU. Phase 1 to 4 are lockdown phases while Phase 5 is the post-lockdown phase. Figures generated using 'Cartopy' version 0.16 and 'Rasterio' version 1.2 modules of Python 3.6.

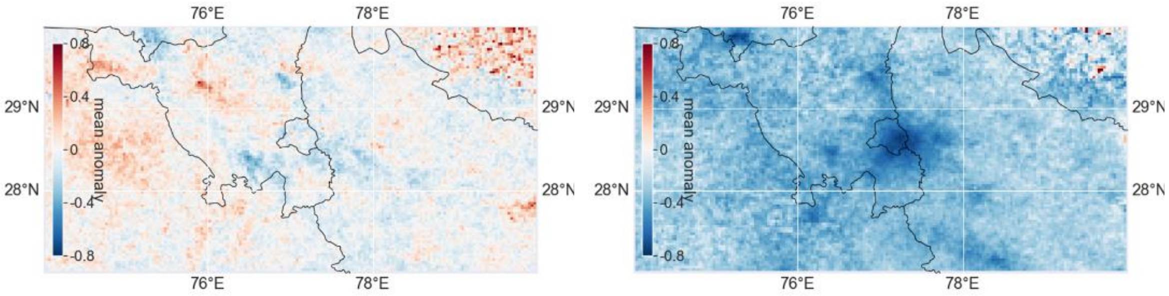

(a) BAU (b) Phase 1

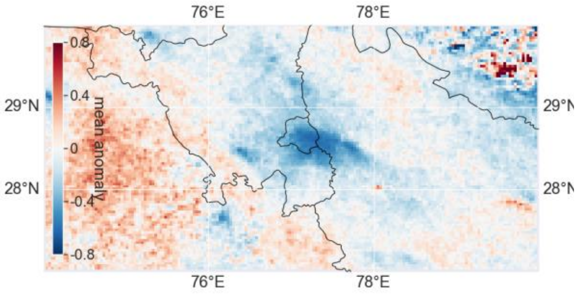

(c) Phase 2

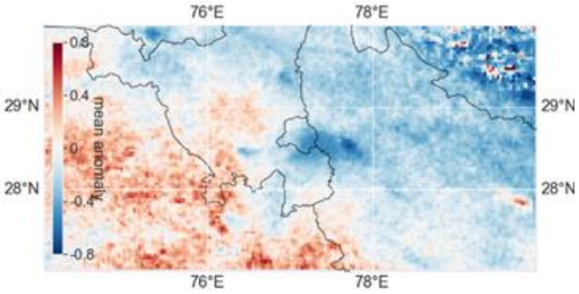

(d) Phase 3

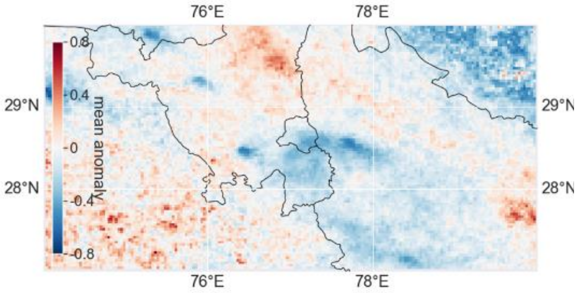

(e) Phase 4

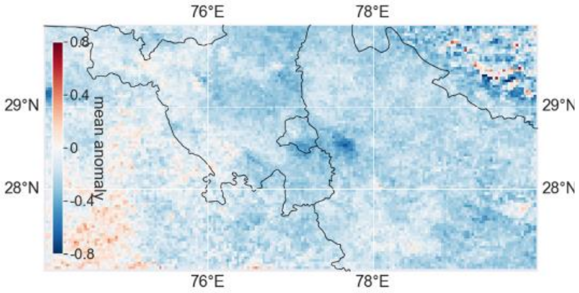

(f) Phase 5

Figure S8. Lockdown phase-wise mean anomaly of TROPOMI NO_2 columns in 2020 with respect to 2019. Pre-lockdown business-as-usual phase is denoted as BAU. Phase 1 to 4 are lockdown phases while Phase 5 is the post-lockdown phase. Figures generated using 'Cartopy' version 0.16 and 'Rasterio' version 1.2 modules of Python 3.6.

S5. *Biomass-fire*

Wheat crop-residue burning in the region is an annual activity that takes place around the month May and June[8]. Figure S7 shows the monthly crop-residue events from the NASA's Fire Information for Resource Management System (FIRMS).

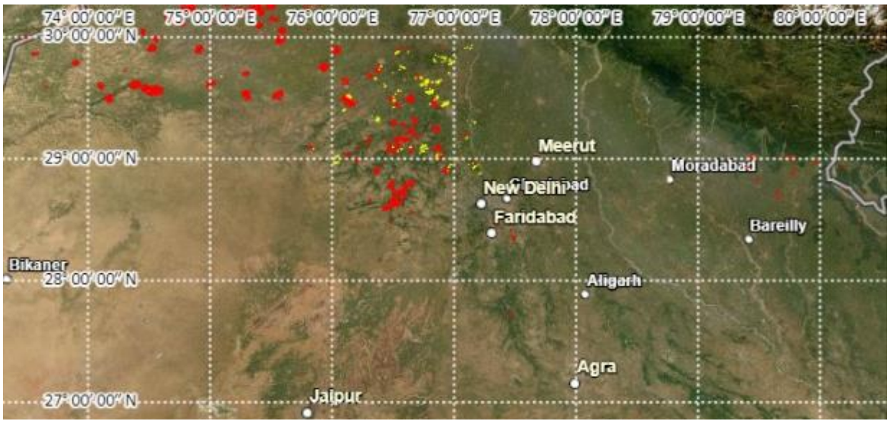

Figure S9. Monthly aggregate biomass fires in May (red) and June (yellow). We acknowledge the use of imagery from the NASA FIRMS application (https://firms.modaps.eosdis.nasa.gov/) operated by the NASA/Goddard Space Flight Center Earth Science Data and Information System (ESDIS) project.

S6. *Top-down NOx emission estimation*

The mean top-down NOx emission rate inventory for the BAU is shown in Figure S10. The total emission rate in the 300×600 km^2 domain was 18.34 kg/sec that corresponds to 1585.16 metric tons/day (by assuming constant diurnal emission rate). In 2020, the 60×60 km^2 region around central Delhi (not including Dadri power-plant) alone accounted for 2.78 kg/sec or 240.19 tons/day NOx emission. The same region in 2019 emitted 3.37 kg/sec or 291.16 tons/day. Spatially local peaks of the emission were found within 3 pixels (10 km) of the emission sources such as power-plants[PM1]. Emission clusters corresponding to strongly emitting power-plants, factories, highways are identifiable during BAU. Especially highways that radiate from Delhi have higher NOx emission due to vehicular traffic as well as industrial clusters along highways.

Bottom-up and regional NOx emission inventories are often found to have been overestimated [PM2]compared to the satellite based top-down emission estimation, such as in Delhi[5] and Germany[9]. For the year 2000, Gurjar et al.,[10] estimated bottom-up NOx emissions to be 441 tons/day. For the year 2010, Sahu et al.,[29] estimated total emissions in a 70×65 km^2 domain size over Delhi as 698 tons/day. Sindhwani et al.,[30] estimated total NOx emission as 937 tons/day in 70×70 km^2 domain while Guttikunda & Calori[31] estimated NOx emission as 1030 tons/day 80×80 km^2 domain. The relative order of emission sources in their contributions as reported by Sindhwani et al.,[30] and Guttikunda & Calori [31] is consistent amongst transport sector (47% to 53%), and diesel generators (25% to 27%) and power plants and industries (11% to 20%). Within Delhi, transport sector alone was found to contribute 54% towards total NOx emissions (161 kt/yr) (Sindhwani et al.,[30]. A reason for a higher emission estimation could be that the above-mentioned bottom-up studies report annual averages that did not consider seasonality of emissions. For example, power-failure leads to a greater use of diesel generators in Delhi during summers, hence higher diesel generator related emissions are during summer than winter. Non-consideration of such seasonal variation in emission may partially explain top-down emission estimation being lower than bottom-up emission inventory.

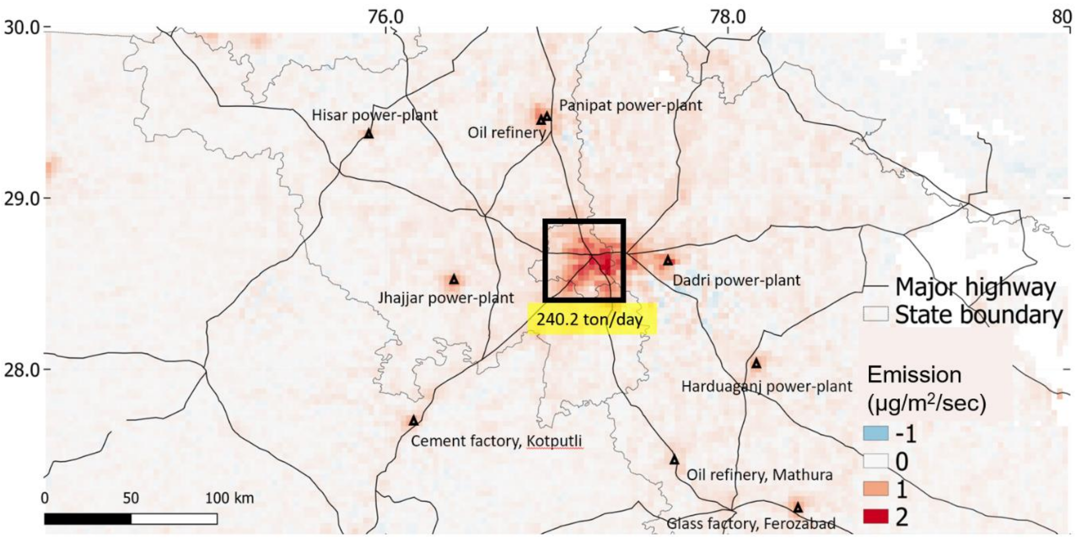

Figure S10. Mean top-down emission rate during business-as-usual case (6 January, 2020 to 24 March, 2020) overlaid with highway-road network map. Strong emission sources are located within the urban center and along major highways and power-plants. Figures generated using 'Cartopy' version 0.16 and 'Rasterio' version 1.2 modules of Python 3.6.

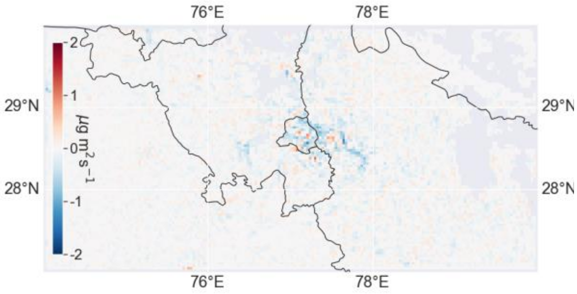

(a) BAU

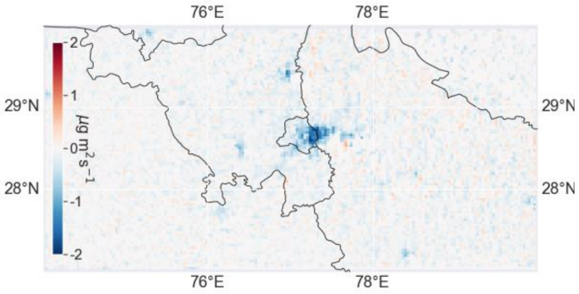

(b) Phase 1

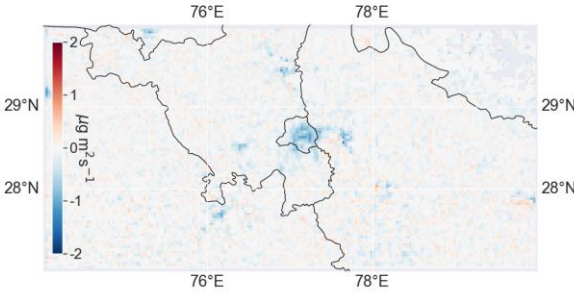

(c) Phase 2

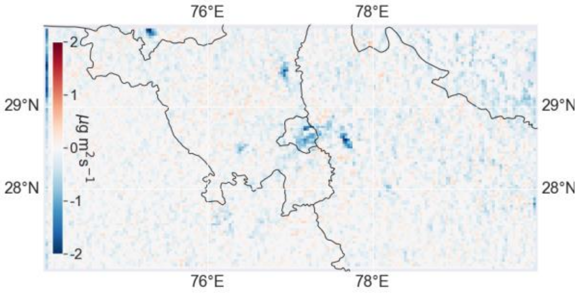

(d) Phase 3

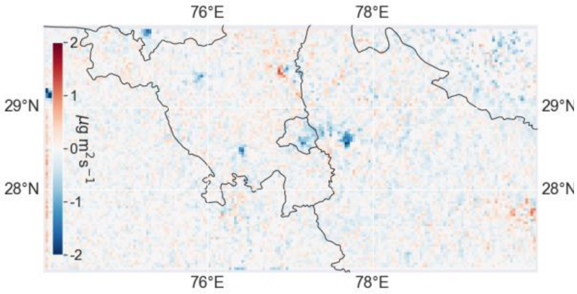

(e) Phase 4

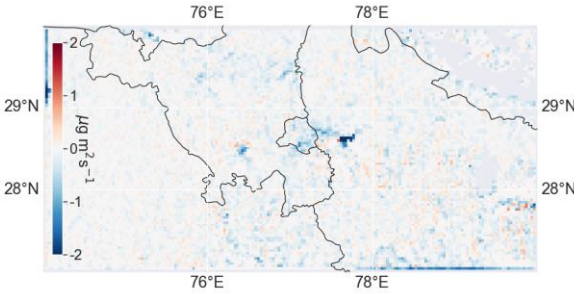

(f) Phase 5

Figure S11. Difference of NOx emission in 2020 and 2019 during (a) BAU (business-as-usual), and subsequent phases of lockdown (b)-(f). Phases are described in Table S1. Figures generated using 'Cartopy' version 0.16 and 'Rasterio' version 1.2 modules of Python 3.6.

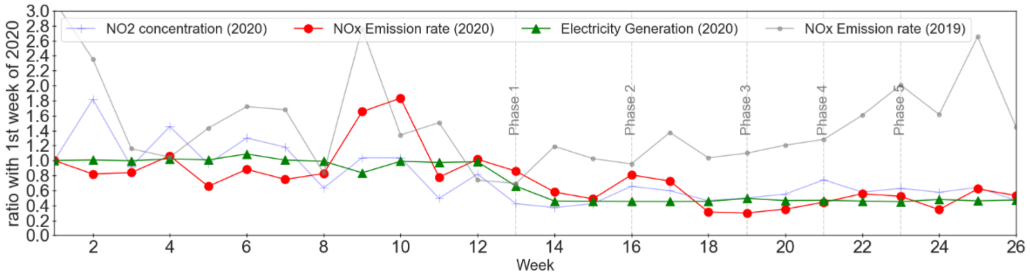

(a) Dadri power plant

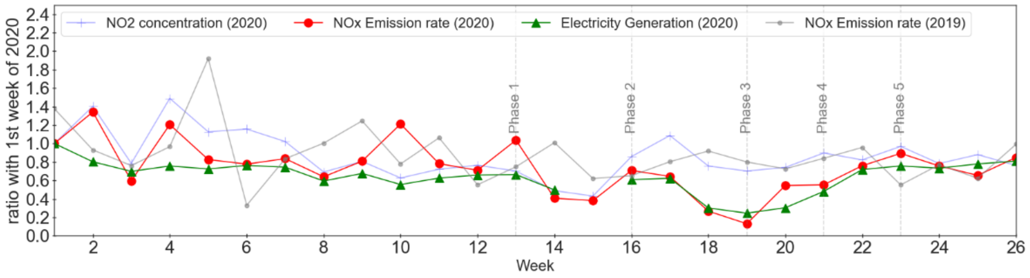

(b) Harduaganj power plant

Figure S12. Mean relative activity, NOx emission and NO_2 concentration with respect to mean values in the first week of January 2020 (6 January – 12 January, 2020) for the power-plants at Dadri and Harduaganj.

Table S2. Pairwise correlations between weekly tropospheric NO_2 concentration, top-down NOx emission rate and reported activity for urban New Delhi and power plants at Dadri and Harduaganj. Activity for urban New Delhi refers to percentage reduction in mobility while activity for power plants refers to electricity generated

location	concentration & emission	concentration & activity	emission & activity
New Delhi (urban)	0.90	0.89	0.96
Dadri	0.64	0.73	0.73
Harduaganj	0.67	0.48	0.63

S7. *Emission uncertainties*

Top-down emissions were calculated via $E = S + D = E = LC/\tau + \nabla(LCw)$. Due to the uncertainties in the datasets used and assumptions, the emission estimates have uncertainties.

Tropospheric NO_2 retrievals suffer uncertainty in slant column density (due to measurement noise and spectral fitting errors), stratospheric slant column (due to error in separating stratospheric and tropospheric NO_2) and tropospheric AMF (due to model parameter errors such as assumed profile shape[14]). The trace gas vertical profile is needed to derive VCD by separating AMF from SCD. Column uncertainty due to AMF is about 30%[14]. If the satellite retrieval assumed NO_2 height profile has a smaller aerosol fraction close to surface compared to the true profile, then tropospheric AMF will be overestimated and correspondingly the retrieved tropospheric NO_2 VCD will be underestimated[14]. Shaiganfar et al., (2011) compared OMI NO_2 VCD with MAX-DOAS observations over Delhi and found that OMI retrievals underestimate high concentrations. Over highly polluted regions tropospheric NO_2 VCDs are partially underestimated due to shielding of emitted NO_2 by aerosols[5]. The random errors are reduced by temporal and spatial averaging, while a major part of systematic errors is expected to cancel out through the difference and ratio in the defined anomaly metric.

Estimating *D* (divergence) and *S* (sink) is complicated mainly by the variation in wind fields and chemical transformation[9,15] respectively, in addition to the tropospheric NO_2 retrieval. As long as the wind has a constant speed and direction and these parameters are known with a high certainty, the steady-state assumption can be applied. [PM3]However slow winds which change directions with time and space complicate emission estimation as such scenarios have high uncertainty (relative to wind speed) and the sudden change in wind direction breaks the steady-state assumption. However, the error due to non-stationary state is smaller near point sources, which is further diminished by taking a multi-temporal mean. The NCEP modeled winds have an uncertainty of about 20% in speed and 14° in direction[16]. Based on the sensitivity analysis by Beirle et al.[9], the speed and direction uncertainty would lead to an uncertainty of 15% and a systematic low bias of 3% respectively in *D*. Another problem is that while the wind speeds and directions change with altitude, the vertical profile of the trace gas itself is not well-known. For example, the near-surface emissions (from vehicles) and chimney-stack emissions are injected at different altitude and their dispersion is subjected to different wind speeds. The vertical levels chosen for the wind fields result in an uncertainty of 10% in the corresponding *D*[9]. The total uncertainty due to the wind fields is the quadratic sum of 15% due to wind speed and 10% due to the plume height, amounting to about 20%.

Chemical reaction rates may also add uncertainties in the assumed lifetime and the constant Leighton ratio. As stated earlier NO_2/NO_x ratio is lower when close to the freshly emitting source in space and time, which increases in aged plumes after chemical conversion[17]. Near strong emission sources emitted NO may not be quickly converted to NO_2 if the NO mixing ratios locally exceed those of ozone. The NO/NO_2 steady state is completely achieved only after ambient air has mixed with the emitted plume[5]. Turbulence in wind speed may enhance mixing and conversion of NO to NO_2 near the source or it may disperse NO downwind before it gets converted to NO_2[17]. We assumed a constant *L* (NO_x/NO_2) of 1.32 neglecting possible changes by day of the week. We assessed the uncertainty in assuming a constant ratio using NO and NO2 vertical column densities modeled from March to May, 2018 over Asia as part of another project, 'Japan's Study for Reference Air Quality Modeling' (J-STREAM) [18]. As shown in Figure S13, L is lowest (~1.3) over polluted regions like urban areas (Delhi) and over a power-plant (Harduaganj) and is relatively high (~1.4), slightly decreasing between March to May. Standard deviation over polluted regions is 0.06 or about 5%.

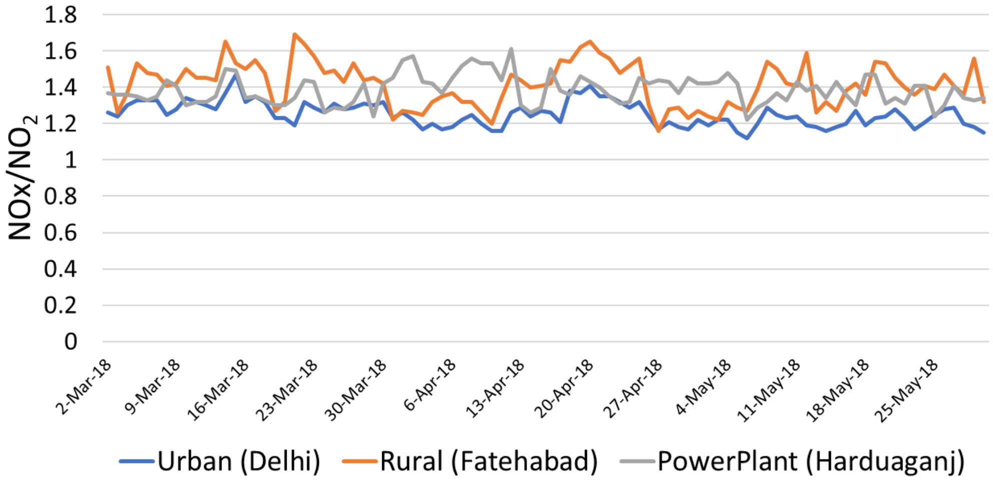

Figure S13. Modeled *L* (NOx/NO_2) vertical column densities over polluted (urban and power-plant) and clean (rural) location.

We assumed that chemical loss of NOx is first-order given by lifetime τ varying from 7 hours in January to 4 hours in July[19]. Such a variation only partially accounts for the changes in τ due to seasonality in actinic flux but neglects the changes due to different ozone concentrations on weekends[20] or during the lockdown[21] and dilution by wind. Uncertainty in this assumption was studied using the method of lifetime fit in which the downwind decay of NO_2 from strong NOx emission sources was used for estimating the lifetime. This method was applied to randomly chosen date for each month between January to June over power-plants and urban areas. The lifetime so calculated were within 1.5 hours of the assumed the lifetime. The associated uncertainties in τ were therefore 30%.

Overall, the uncertainties were added in quadrature and summarized in Table S3. Uncertainties in D dominate over area- and point-based emission such as urban areas and power-plants while uncertainties in S dominate over other regions such as rural-areas.

Table S3. Uncertainties in the individual components for calculating top-down emissions.

	Uncertainty
C (TROPOMI NO2 column)	30%
L (NOx/NO2 ratio)	5%
w (wind speed)	20%
τ (lifetime)	30%
S (sink)	42%
D (divergence)	36%

References

1. Uno, I. *et al.* Systematic analysis of interannual and seasonal variations of model-simulated tropospheric NO2 in Asia and comparison with GOME-satellite data. *Atmospheric Chemistry and Physics* **7**, 1671–1681 (2007).
2. Schenkeveld, V. M. E. *et al.* In-flight performance of the Ozone Monitoring Instrument. *Atmospheric Measurement Techniques* **10**, 1957–1986 (2017).
3. Torres, O., Bhartia, P. K., Jethva, H. & Ahn, C. Impact of the Ozone Monitoring Instrument Row Anomaly on the Long-term Record of Aerosol Products. *Atmos. Meas. Tech. Discuss.* **11**, 2701–2715 (2018).
4. Ghude, S. D., Kulkarni, P. S., Kulkarni, S. H., Fadnavis, S. & Van Der A, R. J. Temporal variation of urban NO $_x$ concentration in India during the past decade as observed from space. *Int. J. Remote Sens.* **32**, 849–861 (2011).
5. Shaiganfar, R. *et al.* Estimation of NO x emissions from Delhi using Car MAX-DOAS observations and comparison with OMI satellite data. *Atmos. Chem. Phys.* **11**, 10871–10887 (2011).
6. Lambert, J. C., Keppens, A., Hubert, D. & Langerock, B. *Sentinel-5 Precursor Mission Performance Centre Quarterly Validation Report of the Sentinel-5 Precursor Operational Data Products # 01 : July – October 2018*. (2020).
7. Nitta, K., Misra, P. & Hayashida, S. Intercomparison of TROPOMI and OMI Tropospheric Nitrogen Dioxide over South Asia (submitted). *Remote Sens.*
8. Venkataraman, C. *et al.* Emissions from open biomass burning in India: Integrating the inventory approach with high-resolution Moderate Resolution Imaging Spectroradiometer (MODIS) active-fire and land cover data. *Global Biogeochem. Cycles* **20**, 1–12 (2006).
9. Beirle, S. *et al.* Pinpointing nitrogen oxide emissions from space. *Sci. Adv.* **5**, 1–7 (2019).
10. Gurjar, B. R., Van Aardenne, J. A., Lelieveld, J. & Mohan, M. Emission estimates and trends (1990-2000) for megacity Delhi and implications. *Atmospheric Environment* **38**, 5663–5681 (2004).
11. Sahu, S. K. High Resolution Emission Inventory of NOx and CO for Mega City Delhi, India. *Aerosol Air Qual. Res.* **2015**, 1137–1144 (2015).
12. Sindhwani, R., Goyal, P., Kumar, S. & Kumar, A. Anthropogenic emission inventory of criteria air pollutants of an urban agglomeration - National capital region (NCR), Delhi. *Aerosol Air Qual. Res.* **15**, 1681–1697 (2015).
13. Guttikunda, S. K. & Calori, G. A GIS based emissions inventory at 1 km x 1 km spatial resolution for air pollution analysis in Delhi, India. *Atmos. Environ.* **67**, 101–111 (2013).
14. Boersma, K. F., Eskes, H. J. & Brinksma, E. J. Error analysis for tropospheric NO2 retrieval from space. *J. Geophys. Res. Atmos.* **109**, n/a-n/a (2004).
15. Ibrahim, O. *et al.* Car MAX-DOAS measurements around entire cities: Quantification of NOx emissions from the cities of Mannheim and Ludwigshafen (Germany). *Atmos. Meas. Tech.* **3**, 709–721 (2010).
16. Asher, E. *et al.* Novel approaches to improve estimates of short-lived halocarbon emissions during summer from the Southern Ocean using airborne observations. *Atmos. Chem. Phys.* **19**, 14071–14090 (2019).
17. Kimbrough, S., Chris Owen, R., Snyder, M. & Richmond-Bryant, J. NO to NO2 conversion rate

analysis and implications for dispersion model chemistry methods using Las Vegas, Nevada near-road field measurements. *Atmospheric Environment* **165**, 23–34 (2017).

18. Chatani, S. *et al.* Overview of model inter-comparison in Japan's study for reference air quality modeling (J-STREAM). *Atmosphere (Basel).* **9**, (2018).
19. Martin, R. V. Global inventory of nitrogen oxide emissions constrained by space-based observations of NO 2 columns. *J. Geophys. Res.* **108**, 4537 (2003).
20. Beirle, S., Platt, U., Wenig, M. & Wagner, T. Weekly cycle of NO2 by GOME measurements: A signature of anthropogenic sources. *Atmospheric Chemistry and Physics* **3**, 2225–2232 (2003).
21. Mahato, S., Pal, S. & Ghosh, K. G. Effect of lockdown amid COVID-19 pandemic on air quality of the megacity Delhi, India. *Sci. Total Environ.* **730**, 139086 (2020).
